# ﻿Aquatic macrophytes of Angola: a preliminary verified checklist

**DOI:** 10.3897/phytokeys.259.147785

**Published:** 2025-07-04

**Authors:** Rafael Somandjinga, Sílvia Quadros, Francisco Maiato, David Goyder

**Affiliations:** 1 CIBIO, Research Centre in Biodiversity and Genetic Resources, InBio Associate Laboratory, BIOPOLIS Program in Genomics, Biodiversity and Land Planning; UNESCO Chair – Land Within Sea: Biodiversity & Sustainability in Atlantic Islands, University of the Azores, Rua da Mãe de Deus, 9500-321 Ponta Delgada, Portugal; 2 Faculty of Science and Technology, University of the Azores, Azores, Portugal; 3 Faculty of Natural Sciences, University of Namibe, Farol de Noronha Campus 274, Moçâmedes, Namibe, Angola; 4 Mandume Ya Ndemufayo University, Avenida Hoji Ya Henda, 30, Lubango, Angola; 5 Herbário do Lubango, ISCED-Huíla, Rua Sarmento Rodrigues S/N, Lubango, Angola; 6 cE3c - Centre for Ecology, Evolution and Environmental Changes, Faculty of Sciences, University of Lisbon, Lisboa, Portugal; 7 Royal Botanic Gardens, Kew, Richmond, Surrey, TW9 3AE, London, UK

**Keywords:** Angola, aquatic macrophytes, conservation status, distribution

## Abstract

Aquatic macrophytes are photosynthetic macroscopic organisms that grow permanently or periodically submerged or floating on the surface of the water. The aim of this study is to compile a checklist of fresh-water aquatic vascular plant macrophytes occurring in Angola, focusing on their origins, life forms, conservation status and distribution throughout the country. The checklist was compiled through a literature review of previous studies carried out in Angola, herbarium collections and online databases. A total of 526 species of macrophytes in 196 genera and 70 families was recorded. Cyperaceae was the most abundant family, followed by Poaceae, Lentibulariaceae, Lythraceae, Eriocaulaceae, Araceae, Podostemaceae, Hydrocharitaceae, Onagraceae and Plantaginaceae. The highest number of macrophytes was recorded from Huíla Province, followed by Moxico and Cuando Cubango Provinces, respectively, while Lunda Norte, Uíge and Zaire were poorly represented. From the aquatic macrophytes recorded, it was possible to identify the category of origin of 511 species. From these, 472 species are native, 23 introduced and 16 are endemic to Angola and the conservation status of these species was also assessed. The results obtained in this study show that there are still some gaps, especially with regard to the distribution of aquatic macrophytes in Angola, since many species are documented as native, but there is a lack of data on their distribution.

## ﻿Introduction

Aquatic macrophytes are macroscopic photosynthetic organisms, that grow permanently or periodically submerged or that float on the water surface (Chambers et al. 2008; Murphy et al. 2019; Kato et al. 2024). These plants have been recognised as a significant component of aquatic ecosystems, which play an important role in providing several environmental and ecosystem services (Sood et al. 2012; Vymazal 2013; Kumar et al. 2023); these include, decontamination and recycling of nutrients in water (Khan et al. 2020; Kumar et al. 2020), control of water erosion (Kumar et al. 2023), biomass production (Amulya et al. 2023; Dębowski et al. 2023), act as organic fertilisers (Saleh 2023; Hernández-Fernández et al. 2024) and as water quality bio-indicators (Shelef et al. 2013; Bytyqi et al. 2020; Saleh 2023). In addition to these ecosystem services, aquatic macrophytes can also be used to perform wastewater treatment in extensive systems, such as constructed wetlands (Vymazal 2013).

Based on the habitat that these groups of plants occupy, macrophytes can be classified as: i) emergent macrophytes - rooted in submerged soils or land that are periodically flooded, with foliage that extends into the air; ii) floating leaf macrophytes - rooted in the bottom of lake or stream with leaves floating on the surface of the water; iii) free-floating macrophytes - plants floating on or under the surface of the water; iv) submerged macrophytes - plants that grows completely submerged under water (Chambers et al. 2008) and v) amphibious macrophytes - plants capable of living both in flooded areas and above water (Moura-Júnior and Cotarelli 2019).

Angola is a country with a rich biodiversity. However, vegetation studies in Angola are limited to individual or few published studies in scientific literature and most of the published literature and/or databases are not easily accessed (Huntley et al. 2019). The scarcity of vegetation studies of Angola was also mentioned by Figueiredo and Smith (2008), Figueiredo et al. (2009), Sosef et al. (2017), Goyder and Gonçalves (2019) and Goyder et al. (2023a). Although some studies have already been carried out, in most of these areas, the focus has been predominantly on the exploration of terrestrial groups (Figueiredo and Smith 2008; Figueiredo et al. 2009; Chisingui et al. 2018; Goyder et al. 2018; Gonçalves et al. 2021; Monteiro et al. 2022; Goyder et al. 2023a, 2023b; Almeida et al. 2024).

From the studies about aquatic macrophytes carried out in Angola, the collections of Hans Hess in early 1950 deserve special mention (Goyder and Gonçalves 2019). Other important work on aquatic macrophytes by Gomes (2009), who presented a vegetation study of the Angolan section of the Okavango Basin, referring to different aquatic macrophytes identified in this section of the Angolan territory, with about 23 species recorded. Goyder et al. (2018) presented a checklist of vascular plants, where they highlighted aquatic macrophytes of the Okavango Basin, stating that the wetlands of the region tend not to be botanically diverse and do not have local endemics. Therefore, although we verified some studies related to aquatic macrophytes, in Angola, there is no complete checklist of this important group of plants.

Compiling data into a checklist, based on fieldwork, bibliographic review, herbarium collections or online data platforms, allows us to increase knowledge related to biodiversity in a given study area, as well as to identify efficient tools to obtain scientific data (Moura-Júnior et al. 2013). This work aims to compile a preliminary verified checklist of the fresh-water aquatic vascular plant macrophytes in Angola, focusing on the category of origin, life form, conservation status and distribution throughout the country.

## ﻿Material and methods

### ﻿Study area

**Figure 1.**
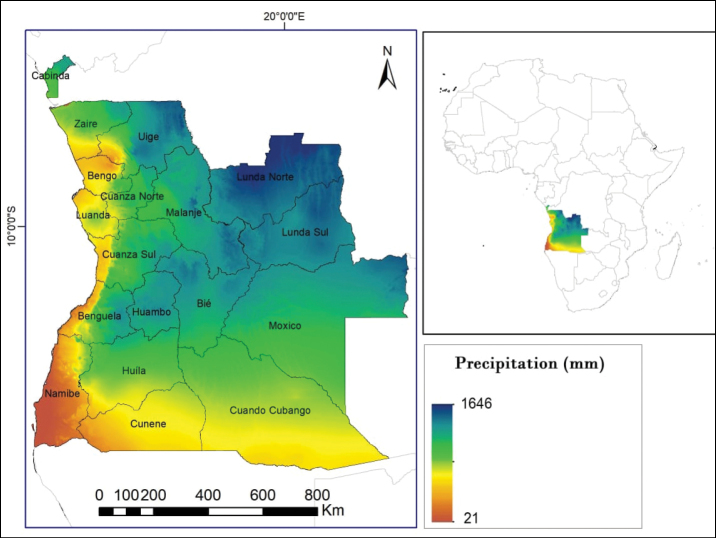
Angola’s geographical location and mean annual precipitation.

Angola is a country with an area of 1,246,700 square kilometres, located on the southwest coast of Africa, between latitude 4°22' and 18°02' South and longitude 11°41' and 24°05' East. It is bordered to the west by the Atlantic Ocean; to the north by the Republic of Congo and the Democratic Republic of Congo (DRC); to the east by the DRC and Zambia; to the south by Namibia (Huntley et al. 2019) (Fig. 1). According to the Koppen-Geiger climate classification, the northern region of Angola is typical of the tropical humid savannah group (Aw), the plateau is typical of the temperate mesothermal group (Cw), the southwest and the coastal plain are typical of the dry desert and semi-desert group (Bsh, Bwh) and the east has a temperate climate with hot summer rains (Cwa) (Huntley et al. 2019). Average annual rainfall varies from 37 mm in the desert province of Namibe to over 1600 mm in the provinces of Uíge, Lunda Norte and Lunda Sul. Angola is a country, rich in water resources; it contains nine major hydrological basins, six of which are transnational, serving as a “water tower” for much of southern and central Africa (Lourenco and Woodborne 2023); this Angolan water tower feeds major tributaries of the Congo, Zambezi and Okavango systems, as well as the Cuanza and Cunene Rivers (Milzow et al. 2009; Abiodun et al. 2019; Huntley et al. 2019; Lourenco et al. 2022).

Angola has been part of the Ramsar Convention since October 2021 (Ramsar 2025; Knight and Lourenco 2025) and, within the framework of the recommendations of this Convention, 11 candidate sites have been identified for inclusion on the list of Ramsar sites, namely: Lagunas do Mangal do Lobito (Benguela), Saco dos Flamingos (Luanda), Lagoa do Arco (Namibe), Parque Nacional de Cameia (Moxico), Complexo das Zonas Húmidas da Lagoa do Carumbo (Lunda Norte), Lagoa do Calumbo (Luanda), Lagoa da Quilunda (Luanda) Complexo de Lagunas de Santiago Saurico (Bengo), Lagoa do Mangal do Chiloango (Cabinda), Baixo Cuanza (Luanda) and Complexo das Zonas Húmidas do Kumbilo Dirico (Cuando Cubango) (INBC 2021; Knight and Lourenco 2025). However, there is very little information on these wetlands and details are not yet available on the convention’s website (Knight and Lourenco 2025). We also realise that, of these 11 wetlands, only two (Parque Nacional de Cameia (Moxico) and Complexo das Zonas Húmidas da Lagoa do Carumbo (Lunda Norte)) are located in the area with the highest average annual rainfall in the country, which indicates the scarcity of studies on wetlands.

In terms of its flora, Angola has around 6850 native plant species, a level of endemism of around 14.8% and 230 introduced species, four of which are considered highly invasive (Rejmánek et al. 2017; Goyder and Gonçalves 2019). According to Zigelski et al. (2019), fires, herbivory, extreme minimum temperatures and frost are factors that play significant roles in the floristic composition and physiognomic structure of the Angolan vegetation.

### ﻿Collection of data

The checklist was drawn-up by conducting a literature review of previous botanical and floristic studies carried out in Angola (Costa et al. 2004; Figueiredo and Smith 2008; Gomes 2009; Goyder et al. 2018; Monteiro et al. 2022; Goyder et al. 2023b) and in the specialised literature in aquatic macrophytes (Fischer 1997; Germishuizen and Meyer 2003; Van Ginkel et al. 2011; Moura-Júnior et al. 2015; Cheek et al. 2017; Sieben et al. 2017; Ameka and Ernest 2019; Murphy et al. 2019; Pestana et al. 2024). Online databases, such as the International Union for the Conservation of Nature-Red list (IUCN 2024), Plants of the World Online (POWO 2024) and Global Biodiversity Information Facility (GBIF 2024) were also used. We also used the aquatic macrophyte collection of the Herbarium of Lubango (LUBA), housed at ISCED-Huíla, where we extracted the vouchers information from the specimens. Plant names were standardised by reference to POWO (2024) for Angiosperms, which incorporates the family concepts of APG IV (Angiosperm Phylogeny Group IV 2016) and Roux (2009) for Pteridophytes and Lycophytes. The species were classified according to life form, based on Gomes (2009), Moura-Júnior et al. (2015), Ameka and Ernest (2019), Moura-Júnior and Cotarelli (2019) and Pestana et al. (2024). The species were recorded by province, the codes for the Angolan provinces being based on Figueiredo and Smith (2008). For category of origin we used the terminology: native, endemic and introduced, also based on Figueiredo and Smith (2008) and POWO (2024). The conservation status of the species: Data Deficient (DD), Least Concern (LC), Near Threatened (NT), Vulnerable (VU), Endangered (EN) and Critically Endangered (CR) uses the IUCN (2024) categories and criteria.

## ﻿Results

### ﻿Diversity

**Figure 2.**
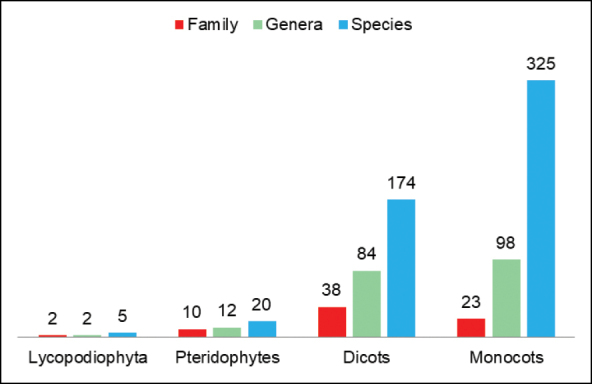
Distribution of the number of species, genera and families for each category (Lycopodiophyta, Pteridophytes, Dicots and Monocots).

A total of 526 species were registered, these belonging to 196 genera and 70 botanical families of aquatic macrophytes. The species were grouped according to major classification of plants: lycophytes (5 species), pteridophytes (20 species), dicots (176 species) and monocots (325 species) (Fig. 2). The botanical families with major number of species were Cyperaceae (155 spp.), Poaceae (70 spp.), Lentibulariaceae (21 spp.), Lythraceae (18 spp.), Eriocaulaceae (17 spp.), Podostemaceae (14 spp.), Araceae and Hydrocharitaceae (11 spp.) and Onagraceae and Plantaginaceae (10 spp.) (Table 1). These ten families together account for 60% of the total species recorded. Of all the species recorded in this study, 174 are deposited at LUBA.

**Table 1.** Number of species (richness) distributed according to respective botanical family.

Family	Number of species
**Cyperaceae**	155
**Poaceae**	70
**Lentibulariaceae**	21
**Lythraceae**	18
**Eriocaulaceae**	17
**Podostemaceae**	14
**Araceae, Hydrocharitaceae**	11
**Onagraceae, Plantaginaceae**	10
**Asteraceae, Fabaceae, Xyridaceae**	9
**Marsileaceae, Orchidaceae**	8
**Alismataceae, Commelinaceae, Polygonaceae**	7
**Droseraceae**	6
**Linderniaceae, Menyanthaceae, Nymphaeaceae, Pontederiaceae, Potamogetonaceae, Thelypteridaceae, Lamiaceae**	5
**Orobanchaceae, Araliaceae, Boraginaceae, Apocynaceae, Apiaceae**	4
**Aponogetonaceae, Convolvulaceae, Hydrostachyaceae, Juncaceae, Lycopodiaceae, Melastomaceae**	3
**Amaryllidaceae, Acanthaceae, Ceratophyllaceae, Gentianaceae, Haloragaceae, Iridaceae, Isoetaceae, Juncaginaceae, Dryopteridaceae, Ranunculaceae, Typhaceae, Rubiaceae, Parkeriaceae**	2
**Cannaceae, Amaranthaceae, Azollaceae, Brassicaceae, Cabombaceae, Celastraceae, Colchicaceae, Cymodoceaceae, Elatinaceae, Equisetaceae, Gleicheniaceae, Theophrastaceae, Ruppiaceae, Primulaceae, Ochnaceae, Mayacaceae, Marantaceae, Hypericaceae, Sphenocleaceae, Malvaceae**	1

**Figure 3.**
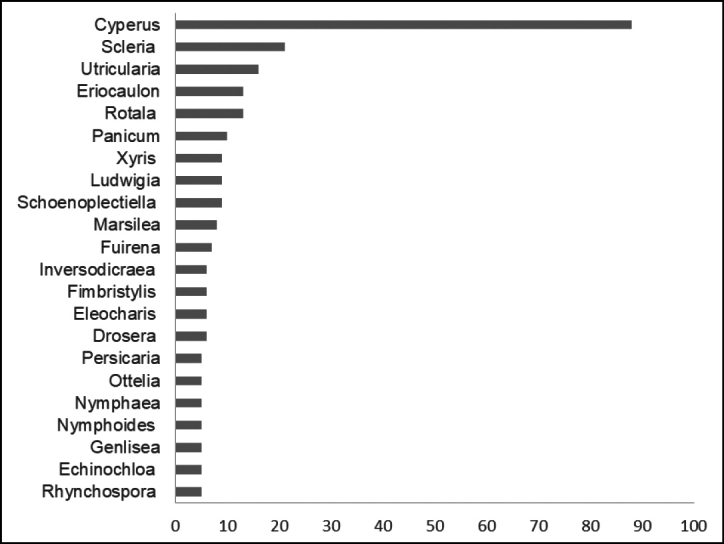
Distribution of species by genera.

The genera with the highest numbers of species are *Cyperus* L. (88), *Scleria* P.J.Bergius (21), *Utricularia* L. (16), *Eriocaulon* L. and *Rotala* L. (13) and *Panicum* L. (10) (Fig. 3). Its important to mention that these genera belong to the five richest families, mentioned above, namely: Cyperaceae (*Cyperus*, *Scleria*,), Lentibulariaceae (*Utricularia*), Eriocaulaceae (*Eriocaulon*), Lythraceae (*Rotala*) and Poaceae (*Panicum*). In contrast, 105 genera were represented by a single species.

### ﻿Distribution of aquatic macrophytes by province

**Figure 4.**
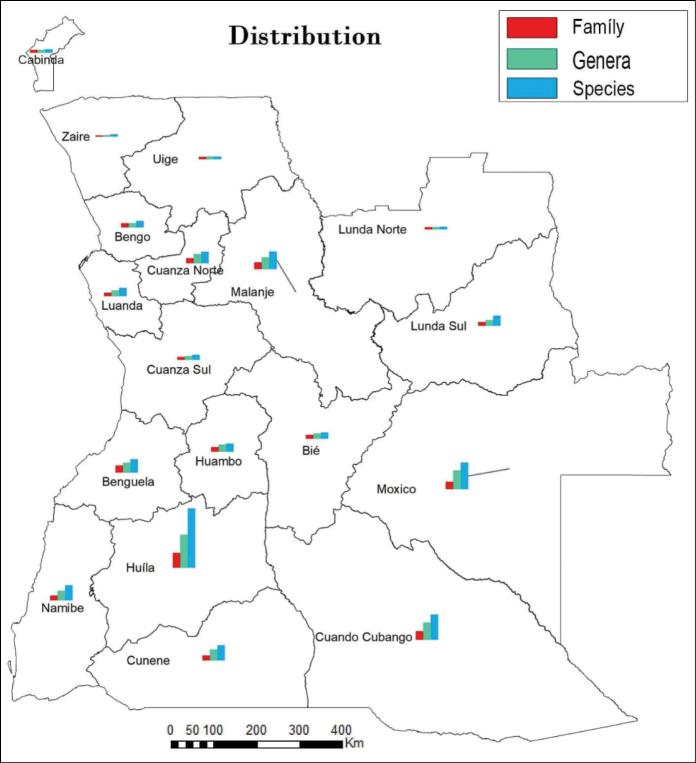
Distribution of aquatic macrophyte by province, showing the high representation of Huíla Province.

The distribution of macrophytes shows that Huíla Province is the best represented with a total 163 species, followed by Moxico and Cuando Cubango, Provinces with 74 and 71 species, respectively. Malanje (49 species), Cunene (43 species) and Namibe (41 species) come next (Fig. 4). The provinces with the least representation are: Lunda Norte, Uige and Zaire with fewer than 10 species. It should be noted that it was not possible to ascertain the distribution of 189 species.

### ﻿Life forms

**Figure 5.**
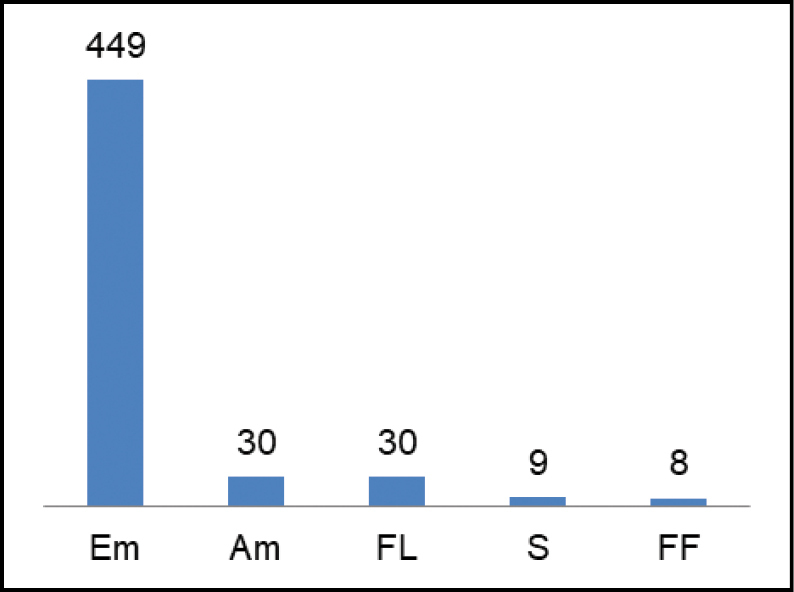
Distribution of species by life form: Em (Emergent), Am (Amphibious), FL (Floating-leaved), S (submersed), FF (Free floating).

As far as biological forms are concerned, the species with the highest number of records were emergent macrophytes, with around 85%, followed by amphibious and floating-leaved macrophytes with around (6%) and finally free-floating and submerged macrophytes with around 2% (Fig. 5).

### ﻿Category of origin of aquatic macrophytes

**Figure 6.**
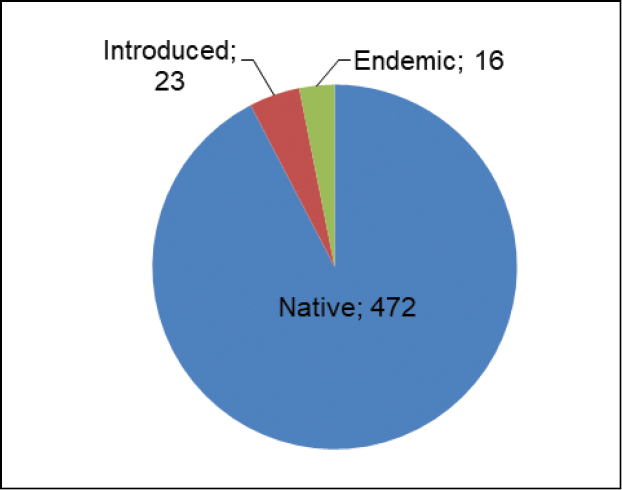
Category of origin of the aquatic macrophytes recorded from this study.

From the recorded aquatic macrophytes, it was possible to identify the category of origin of 511 species. From these, 472 species are native, corresponding to 91%, 23 especies are introduced, representing 4% and 16 are endemic to Angola, corresponding 3% (Fig. 6). The most representative, in terms of endemism are Cyperaceae (4 spp.), (*Cyperusgossweileri* Kük., *Cyperussubtenax* Kük., *Eleochariscubangensis* H.E.Hess, *Scleriapulchella* Ridl.) and Lythraceae with three spp. (*Rotalanummularia* Welw. ex Hiern, *R.thymoides* Exell, *Dissotisrhinanthifolia* (Brenan) A.Fern. & R.Fern.). The Poaceae family with five spp. (*Arundodonax* L, *Coixlacryma-jobi* L., *Paspalumconjugatum* P.J. Bergius, *P.urvillei* Steud., *Polypogonviridis* (Gouan) Breistr) represents the largest number of introduced species. It was not possible to identify the category of origin of 12 species (*Berulaerecta* (Huds.) Coville, *Genliseaglandulosissima* R.E.Fr., *Nymphoidesaquatica* (J.F. Gmel.) Kuntze, Nymphoidesindica(L.)Kuntzesubsp.occidentalis A.Raynal, *Micrargeriellaaphylla* R.E.Fr., *Aponogetonabyssinicus* Hochst. ex A.Rich., *Cyperussubtrigonus* (C.B.Clarke) Kük., FuirenaleptostachyaOliv.var.nudiflora K.Schum., *Hypolytrumheterophyllum* Boeckeler, *Brachycorythiscongoensis* Kraenzl., *Disaochrostachya* Rchb. f., *Potamogetonpolygonifolius* Pourr.) and three are doubtful (*Myriophyllumspicatum* L., *Sphenocleazeylanica* Gaertn., *Potamogetonrichardii* Solms).

### ﻿Conservation status

This study recorded a total of 386 species (73%) included in the IUCN Red List (IUCN 2024) from which 366 were considered Least Concern (LC), nine were Data Deficient (DD), (*Droseraelongata* Exell & J.R.Laundon, *Hydrostachysinsignis* Mildbr. & Reimers, *Genliseaglandulosissima* R.E.Fr., *Rotalafontinalis* Hiern, *Nymphaeadivaricata* Hutch., *N.sulphurea* Gilg, *Eleochariscubangensis* H.E.Hess, *Eriocaulonlongipetalum* Rendle, *Xyrisimitatrix* Malme.), six were Vulnerable (VU) (*Utriculariainflexa* Forssk., *U.stellaris* L.f., *Rotalasmithii* A.Fern. & Diniz, *Inversodicraeacristata* Engl., *I.warmingiana* (Gilg) Engl. and *Ledermanniellaaloides* (Engl.) C.Cusset), two Near Threatened (NT) (*Ammanniabaccifera* L. and *Anagalliskochii* H.E.Hess), two Endangered (EN) (*Genliseaangolensis* R.D.Good and *Leiothylaxquangensis* (Engl.) Warm.) and one Critically Endangered (CR) (*Scleriapulchella* Ridl.). The family Podostemaceae was the family with major number of species with Vulnerable status (VU), namely: *Inversodicraeacristata* Engl., *I.warmingiana* (Gilg) Engl. and *Ledermanniellaaloides* (Engl.) C.Cusset. A total of 139 species are not listed on the IUCN Red List.

## ﻿Discussion

### ﻿Diversity

Globally, 3499 species of aquatic macrophytes are known (Lobato-de Magalhães et al. 2023; Lobato-de Magalhães et al. 2024), distributed in six main ecozones (Nearctic, Neotropics, Australasia, Palaearctic, Afrotropics, Orient). Angola is part of the Afrotropics ecozone, which includes a total of 916 species according to Lobato-de Magalhães et al. (2024). In this study, a total of 526 species of aquatic macrophytes were recorded throughout the country, representing aproximatelly 8% of a total of ca. 6850 plant species actually known from Angola (Goyder and Gonçalves 2019).

The total number of aquatic macrophytes recorded, represented mainly by Cyperaceae and Poaceae, were also reported in various studies (Moura-Júnior et al. 2015; Adams et al. 2016; Sieben et al. 2017; Ameka and Ernest 2019; Murphy et al. 2019; Córdova et al. 2022; Pestana et al. 2024). The success of these species in aquatic environments is maybe due to several factors, namely: rapid vegetative and sexual reproduction, which leads to a rapid increase in the population, the ability to regenerate from fragments, high phenotypic plasticity, as well as efficient dispersal mechanisms (Hill et al. 2020).

### ﻿Distribution of aquatic macrophytes

Based on Angola’s varied climatic zones, the various habitat types and the number of macrophytes distributed throughout the country, we can predict that the total number of species recorded is far from the potential we might expect from this vast territory. It should be emphasised that the provinces with the highest number of species in this study are not necessarily the richest and most diverse as shown in Fig. 4. This is because these provinces were more comprehensively documented botanically compared to others, due to many historical reasons and existence of research infrastructures, a colonial legacy, emphasising the need for more and continued studies in other unexplored areas of the country, especially in the provinces located in northern and eastern parts of the country. According to Sieben et al. (2021), there is a strong correlation between the relative number of species and the average annual rainfall. Therefore, these areas represent the Angolan provinces characterised by the highest average annual rainfall (> 1600 mm/yr), so it is to be expected that the number of aquatic macrophytes can be higher than that mentioned in the scientific literature. Another curious fact is related to Namibe Province, characterised with average rainfall (< 600/yr), being occupied mostly by the Namib Desert. According to these characteristics, a low number of aquatic macrophytes would be expected. However, this Province has a considerable number (41) of species. This study was based on the list presented by Figueiredo and Smith (2008), where many documented species do not show their distribution throughout the country and online databases also lack this information. Therefore, given this scenario, the disparity in terms of macrophyte distribution compared to other parts of the country is essentially related to greater effort in floristic surveys in southern parts, when compared to others and, on the other hand, due to the lack of information in databases about the distribution of many species of aquatic macrophytes in Angola.

### ﻿Life form

The most predominant life forms of macrophytes in our study were the emergent species, which can be explained by the ability of these plants to colonise a wide range of aquatic environments, from the beds themselves to the banks and to withstand long periods of drought (Hill et al. 2020). The correct recording of life forms is a fundamental aspect in the study of aquatic macrophytes (Moura-Júnior and Cotarelli 2019). However, this task has often become a major challenge since, in many cases, the labels on collections, herbarium records and some online platforms do not specify or adequately describe the life form and/or habitat, making it difficult to characterise this group of plants (Córdova et al. 2022). Another point that deserves special attention is the inclusion of amphibian species in aquatic macrophyte inventories, since, in some cases, errors can be made due to difficulties in recognising the physical boundary between wet and dry areas. Additionally, on the other hand, due to environmental changes that can occur days before sampling in the field, such as the rapid rise in water levels in an ecosystem due to heavy rainfall (Moura-Júnior and Cotarelli 2019).

### ﻿Category of origin of aquatic macrophytes

In terms of origin categories, the number of endemic species recorded was 16, which represents 3% of total number of species recorded. However, this number may be higher, since classification doubts arose with macrophytes of some endemic genera. It is worth mentioning here some species that are referred to in literature as invasive in various parts of the world and which are recorded in this study, namely *Pontederiacrassipes* Mart. (Datta et al. 2021; Ghoussein et al. 2023), *Pistiastratiotes* L. (Ameka and Ernest 2019; Hill et al. 2020) *Mimosapigra* L. (Shanungu 2009; Welgama et al. 2022), *Cyperuspapyrus* L. (Ameka and Ernest 2019), *Vossiacuspidata* Griff. (Ameka and Ernest 2019; Mahmoud et al. 2021), *Typhadomingensis* Persoon (Edegbene 2018; Ameka and Ernest 2019) *Phragmitesaustralis* (Cav.) Steud (Rohal et al. 2019; Coleman et al. 2023), *Arundodonax* L. (Hardion et al. 2014; Hardesty-Moore et al. 2020; Jiménez-Ruiz et al. 2021; Míguez et al. 2022). A study on invasive plants in Angola, conducted by Rejmánek et al. (2017), points to two of the invasive species listed above (*Pontederiacrassipes* Mart. and *Arundodonax* L.), the latter of which has been spotted in five of the 14 vegetation types in western Angola, more than once and in dense stands. Since these plants cause disturbances in aquatic ecosystems, research should be carried out to understand the behavioural patterns of these species in Angola and, therefore, draw up control and monitoring programmes to guarantee the recovery of the targeted ecosystems.

### ﻿Conservation status of species

The vast majority (95%) of the species assessed were considered to be of Least Concern. However, four species stand out here: one listed as Critical Endangered (CR) (*Scleriapulchella* Ridl.) which, according to Larridon and Bauters (2020), has only been recorded three times, the last being in 1952, near the Humpata and Palanca Rivers in Huíla Province. Three others classified as Endangered (EN) are: *Saxicolellaangola* Cheek which was classified as endangered by Cheek et al. (2022) according to IUCN category and criterion B2ab(iii) (2012), *Genliseaangolensis* R.D.Good considered as a rare species, listed as endangered due to various current threats that are causing a decrease in its habitat and *Leiothylaxquangensis* (Engl.) Warm. which can be found in Cameroon, Angola and the Democratic Republic of Congo (DRC); however, its distribution in Angola is unknown (Ghogue 2010a; Ghogue 2010b). It is worth mentioning a species of the Podostemaceae family (*Angolaeafluitans* Wedd.) which, although not formally evaluated, Goyder and Gonçalves (2019) suggest it may be extinct due to the construction of hydroelectric dams along the Cuanza River.

The species considered vulnerable include: *Rotalasmithii* A.Fern. & Diniz known only from the DRC and Angola. It has been recorded in only two locations in the DRC and a similar distribution is expected to occur also in Angola. This species is threatened by water pollution (Ghogue 2010c). *Inversodicraeacristata* Engl. is known from only a few sites and occupies a much specialised habitat; some of these sites are threatened by habitat disturbance and degradation due to trampling by tourists (Ghogue 2017). *Inversodicraeawarmingiana* (Gilg) Engl. is endemic to southern Angola, in the Cubango River, which then becomes the Okavango River and northern Namibia. Its population is in decline due to environmental degradation (Sieben 2018). *Ledermanniellaaloides* (Engl.) C.Cusset has been recorded globally in only five locations and Angola in only one (Diop 2010). The fact that 140 out of 526 species of aquatic macrophytes are not included on the IUCN Red List is worrying. Research in this area should be encouraged.

## ﻿Conclusions

This study sets a precedent in research into the diversity, distribution, life forms and conservation status of aquatic macrophytes in Angola. Our results show that the Cyperaceae and Poaceae stand out as the most diverse and, in terms of life forms, the category of emergent macrophytes is the most dominant. As for the representativeness of aquatic macrophytes throughout the country, there is an uneven distribution, with the southern provinces being the best documented as a result of the larger floristic surveys that have been carried out.

Native species are the most prevalent, but the presence of introduced and invasive species requires investigation in order to understand the behaviour patterns of these species in Angola and the implementation of continuous monitoring programmes in order to preserve native ecosystems.

The results obtained in this study show that there are still some gaps, especially with regard to the distribution of aquatic macrophytes in Angola, since many species are documented as native, but there is a lack of data on their distribution. In view of the above, we recommend continuing studies on the distribution patterns of aquatic macrophytes in Angola and investing in floristic survey studies in the country’s various provinces, in order to fill in the gaps in knowledge and, thus, promote the preservation of Angola’s aquatic ecosystems.

## ﻿Additional information

### Author ORCIDs

Rafael Somandjinga https://orcid.org/0009-0005-4852-7703

Sílvia Quadros https://orcid.org/0000-0001-8562-6640

Francisco Maiato https://orcid.org/0000-0002-8859-7491

David Goyder https://orcid.org/0000-0002-3449-7313

### Data availability

All of the data that support the findings of this study are available in the main text or Supplementary Information.

## Supplementary materials

Supplementary material 1 Botanical family, category of origin, distribution by province, life form, conservation status, citation and author Data type docx Explanation note A total of 526 aquatic macrophytes species of Angola, listed by their respective botanical family, category of origin, distribution by province, life form, conservation status, citation and author (voucher number). https://binary.pensoft.net/file/1367434This dataset is made available under the Open Database License (http://opendatacommons.org/licenses/odbl/1.0/). The Open Database License (ODbL) is a license agreement intended to allow users to freely share, modify, and use this Dataset while maintaining this same freedom for others, provided that the original source and author(s) are credited.Rafael Somandjinga, Sílvia Quadros, Francisco Maiato, David Goyder
