# Supplementary material for: ﻿Aquatic macrophytes of Angola: a preliminary verified checklist
Source: PhytoKeys. 2025 Jul 4;259:199–215. doi: 10.3897/phytokeys.259.147785 (PMC12254829; doi:10.3897/phytokeys.259.147785)
Supplement: Supplementary material 1 — Botanical family, category of origin, distribution by province, life form, conservation status, citation and author [file phytokeys-259-199_article-147785__-s001.docx]

**Table S1:** Aquatic macrophytes of Angola, listed by their respective botanical family, category of origin, distribution by province, life form, conservation status, citation and author (voucher number). Citations according to the numbers in the table (1) POWO, 2024; (2) IUCN, 2024; (3) Ernest and Ameka, 2019; (4) Roux, 2003; (5) Murphy et al., 2019; (6) LUBA; (7) Goyder et al., 2018; (8) Pestana et al., 2024; (9) Gomes, 2009; (10) Wyk and Tilney, 2003; (11) Victor et al., 2003; (12) Herman et al., 2003; (13) Moura et al., 2015; (14) Glen and Cook, 2003; (15)Bredenkamp, 2003; (16) Retief and Herman, 2003; (17) Smithies, 2003; (18) Smithies and Ready, 2003; (19) Glen and Bredenkamp, 2003; (20) Van Ginkel et al., 2011; (21) Fischer, 1997; (22) Cheek et al., 2017; (23) Germishuizen and Glen, 2003; (24) Welman, 2003; (25) Singh, 2003; (26) Moeng, 2003; (27) Archer, 2003; (28) Goyder et al., 2023b; (29) Cheek et al., 2022; (30) Venter et al., 1990.

| **Family** | **Species** | **Category Of Origin** | **Distribution** | **Life**  **Form** | **Conservation Status** | **Citation** | **Voucher Number** |
| --- | --- | --- | --- | --- | --- | --- | --- |
| **PTERIDOPHYTA** | | | | | | | |
| **Azollaceae** | *Azolla pinnata R.Br.* subsp. *africana* (Desv.) R.M.K.Saunders & K.Fowler | Native | BO CC CN HA MA | FF | LC | 1,2,3,4,5 |  |
| **Dryopteridaceae** | *Bolbitis acrostichoides* (Afzel.) Ching | Native |  | EM |  | 1,4 |  |
|  | *Bolbitis heudelotii* (Bory ex Fée) Alston | Native |  | EM | LC | 1,2,4 |  |
| **Equisetaceae** | *Equisetum ramosissimum* Desf. | Native | CC HI LS NA | EM | LC | 1,2,4,6 | Revermann 135513 |
| **Gleicheniaceae** | *Dicranopteris linearis* (Burm.f.) Underw | Native | HI | EM | LC | 1,2,4,6,7 | Goyder 8396 |
| **Marsileaceae** | *Marsilea coromandelina* Willd. | Native | BE BO | EM | LC | 1,2,4,5 |  |
|  | *Marsilea ephippiocarpa* Alston | Native | HI | EM | LC | 1,2 | Bester et al., 249 |
|  | *Marsilea farinosa* Launert | Native | HI NA | EM | LC | 1,2,4,5 |  |
|  | *Marsilea gibba* A.Braun | Native | CN HI | EM | LC | 1,2,5 |  |
|  | *Marsilea macrocarpa* C.Presl | Native | CN HI MA | EM | LC | 1,2,4,5 |  |
|  | *Marsilea minuta* L. | Native | BO CN HI NA | FL | LC | 1,2,3,4 |  |
|  | *Marsilea nubica* A.Braun | Native | CN CU HI | EM | LC | 1,2,4,5 |  |
|  | *Marsilea unicornis* Launert | Native | CN | EM | LC | 1,4,5 |  |
| **Parkeriaceae** | *Acrostichum aureum* L. | Native | BO UI | EM | LC | 1,2,4 |  |
|  | *Ceratopteris thalictroides* (L.) Brongn. | Native | BO CA | EM | LC | 1,2,4,8 |  |
| **Thelypteridaceae** | *Amauropelta bergiana* (Schltdl.) Holltum | Native | HI | EM | LC | 1,2,4,6 |  |
|  | *Ampelopteris prolifera* (Retz.) Copel. | Native | CN NA | EM |  | 1,4,5 |  |
|  | *Christella dentata* (Forssk.) Brownsey & Jermy | Native | HI | EM | LC | 1,2,4,6 |  |
|  | *Cyclosorus interruptus* (Willd.) H.I | Native | BE BI CN LN MA NA MO | EM | LC | 1,2,4,8 | Goyder 8317 |
|  | *Thelypteris confluens* (Thunb.) C.V.Morton | Native | BI BO CC CN HA HI LS MA | EM | LC | 1,2,4,5,6,9 | Maiato 131; Daniel & Timóteo 3630; Barker et al., 139 |
| **LYCOPODIOPHYTA** | | | | | | | |
| **Isoetaceae** | *Isoetes aequinoctialis* Welw. ex A.Braun | Native | MA | EM | LC | 1,2,4 |  |
|  | *Isoetes welwitschii* A.Braun ex. Kuhn | Native | HI | EM | LC | 1,2,4,5,6 | Daniel & Timóteo 3604 |
| **Lycopodiaceae** | *Lycopodiella affinis* (Bory) Pic.Serm. | Native |  | EM |  | 1,7 | Frisby 3027; Goyder 8261 |
|  | Lycopodiella cernua (L.) Pic.Serm. | Native | HI CS | EM |  | 1,7 | sight record 38; Maiato FM1132; Goyder et al. 8625; Maiato & Camôngua FM1132 |
|  | *Lycopodiella sarcocaulon* (A.Braun & Welw. ex Kuhn) Pic.Serm. | Native |  | EM |  | 7 | Goyder 8298 |
| **DICOTYLEDONS** | | | | | | | |
| **Acanthaceae** | *Hygrophila auriculata* (Schumach.) Heine | Native | HI | EM | LC | 1,2,5 |  |
|  | *Hygrophila lineari*s Burkill | Native |  | EM |  | 1,5 |  |
| **Amaranthaceae** | *Alternanthera sessilis* (L.) R. Br. ex DC. | Introduced | HI | EM | LC | 1,2,3,5,6 | Daniel 3100 |
| **Apiaceae** | *Berula erecta* (Huds.) Coville |  | CN HI | EM | LC | 1,2,5,6,10 | Santos 800; Correia 1266A |
|  | *Berula imbricata*(Schinz) Spalik & S.R.Downie | Native | BE BI CC CS HA LS MO | EM |  | 1,7 | Goyder 8957 |
|  | *Centella asiatica* (L.) Urb. | Native | BE BO CC CN HA HI LA MA NA | EM | LC | 1,2,5 |  |
|  | *Sium repandum* Welw. ex Hiern | Native | CS HA HI NA | EM |  | 1,6,10 | Santos 744 |
| **Apocynaceae** | *Kanahia laniflora* (Forssk.) R.Br. | Native | HI | EM | LC | 1,2,6,11 | Goldsmith 2161; Wakmough 520 |
|  | *Raphionacme linearis* K.Schum | Native |  | EM |  | 1,7 | Goyder & Maiato 8776; Goyder & Maiato 8856 |
|  | *Tacazzea rosmarinifolia* (Decne.) N.E.Br. | Native | CC CU | EM |  | 1,6,30 | Goyder 8064; Goyder & Frisby 8861 |
|  | *Xysmalobium holubii* Scott Elliot | Native | HI | EM |  | 1,7 | Goyder & Maiato 8785; Goyder & Maiato 8853 |
| **Araliaceae** | *Hydrocotyle bonariensis* Comm. ex Lam. | Introduced | CA | EM | LC | 1,2,5,10 |  |
|  | *Hydrocotyle ranunculoides* L.f. | Introduced | MA | EM | LC | 1,2,5,10 |  |
|  | *Hydrocotyle sibthorpioides* Lam. | Native | HI | EM | LC | 1,2,5 |  |
|  | *Hydrocotyle verticillata* Thunb. | Native | BI CC HI MA | EM | LC | 1,2,5,6,10 | Menezes 2398 |
| **Asteraceae** | *Bothriocline monocephala* (Hiern) Wild & G.V.Pope | Native | HI | EM |  | 1,5 |  |
|  | *Cotula coronopifolia* L. | Native |  | EM |  | 1,2,5,12 |  |
|  | *Eclipta prostrata* (L.) L. | Introduced | HI CU | EM | LC | 1,2,5,6,12,13 | Santos 2647; Daniel 3134 |
|  | *Emilia sonchifolia* (L.) DC. ex Wight | Native |  | A |  | 1,13 |  |
|  | *Ethulia conyzoides* L.f. | Native | MO | A | LC | 1,2,6,13 | Finckh & Zigelski 143271 |
|  | *Grangea anthemoides* O.Hoffm. | Native |  | EM |  | 1,5 |  |
|  | *Grangea maderaspatana* (L.) Poir. | Native |  | EM | LC | 1,2,5 |  |
|  | *Senecio strictifolius* Hiern | Native | HI | A |  | 1,6,7 | Miller 7344; Barker et al., 110; Barker et al., 127;  Goyder 8915 |
|  | *Vernonia ornata* S.Moore | Native |  | A |  | 1,7 | Frisby 3091 |
| **Boraginaceae** | *Coldenia procumbens* L. | Native | BO LA | EM | LC | 1,2,5 |  |
|  | *Heliotropium indicum* L. | Introduced | BE LA HI NA | A |  | 1,2,6,8,13 | Menezes 1544; Mendonça 193 |
|  | *Heliotropium ovalifolium* Forssk. | Native | CN LA NA | EM | LC | 1,2,5 |  |
|  | *Heliotropium supinum* L. | Native | NA | EM |  | 1,5 |  |
| **Brassicaceae** | *Nasturtium officinale* R.Br. | Introduced | CN HI | EM | LC | 1,5 |  |
| **Cabombaceae** | *Brasenia schreberi* J.F.Gmel. | Native | CC HI MA MO | FL | LC | 1,2,5,6,7,14 | Goyder 8295 |
| **Celastraceae** | *Gymnosporia senegalensis* (Lam.) Loes. | Native |  | EM |  | 7,29 | Goyder 8934; Maiato et al. FM1796 |
| **Ceratophyllaceae** | *Ceratophyllum demersum* L. | Native |  | S | LC | 1,2,5,6,14 | Ward & Ward 75 |
|  | *Ceratophyllum muricatum* Cham. | Native |  | EM | LC | 1,2,5 |  |
| **Convolvulaceae** | *Ipomoea aquatica* Forsk. | Native | HI CU | EM | LC | 1,2,3,5,6,13 | Gouveia 1033; Menezes 2462 |
|  | *Ipomoea alba* L. | Introduced |  | EM | LC | 1,2,5 |  |
|  | *Ipomoea asarifolia* (Desr.) Roem. & Schult. | Native |  | EM |  | 1,5 |  |
| **Droseraceae** | *Drosera affinis* Welw. ex Oliv | Native | CC HI LS MO | EM | LC | 1,2,6 | Goyder et al., 7472 |
|  | *Drosera bequaertii* Taton | Native | LS | EM | LC | 1,2 |  |
|  | *Drosera burkeana* Planch. | Native | CC HI LS | EM | LC | 1,2,7 |  |
|  | *Drosera elongata* Exell & J.R.Laundon | Native | LS | EM | DD | 1,2 |  |
|  | *Drosera madagascariensis* DC | Native | BE CC HA HI LN LS MA MO | EM | LC | 1,2 |  |
|  | *Drosera pilosa* Exell & J.R.Laundon | Native | CC LS | EM | LC | 1,2 |  |
| **Elatinaceae** | *Bergia ammannioides* Roxb. | Native | CU | EM | LC | 1,2,5,6,15 | Gouveia 1128 |
| **Fabaceae** | *Aeschynomene elaphroxylon* Guill & Perr. | Native | BE | EM | LC | 1,2,3,6 | Patel & Nachamba 3850 |
|  | *Aeschynomene fluitans* Peter | Native | HI | EM |  | 1,5,9 |  |
|  | *Aeschynomene indica* L. | Native | HI CU | EM | LC | 1,2,5 |  |
|  | *Chamaecrista mimosoides* (L.)Greene | Native | HI CC LA | EM | LC | 1,2,6 | Huntley 3485; Revermann 136214 |
|  | Crotalaria pallida Aiton | Native | LA | A |  | 1,6 | Salubeni et al., 4215; Maiato et al. FM1630 |
|  | *Desmodium adscendens* (Sw.) DC. | Native | UI | A | LC | 1,2 |  |
|  | *Mimosa pigra* L. | Introduced | LA CC | EM | LC | 1,2,3,6 | Finckh & Zigelski 143599 |
|  | *Neptunia oleracea* Lour | Introduced | HI CU | EM | LC | 1,2,3,5 |  |
|  | *Vigna luteol*a (Jacq.) Benth. | Native |  | EM | LC | 1,2 |  |
| **Gentianaceae** | *Faroa salutaris* Welw. | Native | BE BI CC HA HI LS MA MO | EM |  | 1,6,7 | Moreno 22; Menezes 1904; Finckh & Zigelski 143547 |
|  | *Neurotheca congolana* De Wild. & T.Durand | Native | BE CC MO | EM |  | 1,7 |  |
| **Haloragaceae** | *Laurembergia tetrandra* (Schott) Kanitz | Native | CC HI LS MA | EM | LC | 1,2,5 |  |
|  | *Myriophyllum spicatum* L. | Doubtful |  | S | LC | 1,2,5 |  |
| **Hydrostachyaceae** | *Hydrostachys insignis* Mildbr. & Reimers | Native | HI | EM | DD | 1,2,5 |  |
|  | *Hydrostachys polymorpha* Klotzsch | Native | BI CU CS | EM | LC | 1,2,5,14 | Gomes visual record |
|  | *Hydrostachys triaxialis* Engl. & Gilg | Native | CC HI | EM |  | 1,5,6 | Bester et al., 41 |
| **Hypericaceae** | *Hypericum oligandrum* Milne-Redh. | Native | BI | EM |  | 1,5 |  |
| **Lamiaceae** | *Coleus betonicifolius* (Baker) A.J.Paton | Native | MO | EM |  | 1,7 | Goyder 8463 |
|  | *Coleus mirabilis* Briq. | Native | MO | EM |  | 1,7 | Barker et al. 140; Baum 794; Goyder 8007; Goyder 8928 |
|  | *Hyptis spicigera* Lam | Introduced |  | EM |  | 1,6 | Kwatha et al., 193 |
|  | *Mentha aquatica* L. | Native | HI | EM | LC | 1,2,5,6,16 | Winter 7639 |
|  | *Platostoma coeruleum (R.E.Fr.) A.J.Paton* | Native |  | EM |  | 1,5 |  |
| **Lentibulariaceae** | *Genlisea africana* Oliv. | Native |  | EM |  | 1,5 |  |
|  | *Genlisea angolensis* R.D.Good | Endemic | MO | EM | EN | 1,2,5,7 |  |
|  | *Genlisea glandulosissima* R.E.Fr. |  |  | EM | DD | 1,2,5 |  |
|  | *Genlisea hispidula*  Stapf | Native |  | EM | LC | 1,2,5,17 |  |
|  | *Genlisea pallida Fromm & P.Taylor* | Native |  | EM |  | 1,5 |  |
|  | *Utricularia andongensis* Welw. ex Hiern | Native | CN UI | EM | LC | 1,2 |  |
|  | *Utricularia arenaria* A.DC. | Native | BE CC HI MA | EM |  | 1,17 |  |
|  | *Utricularia benjaminiana* Oliv. | Native | CC LS MO | FL | LC | 1,2,5,17 |  |
|  | *Utricularia cymbantha* Oliv. | Native | HI MO | EM | LC | 1,2,5,17 |  |
|  | *Utricularia firmula* Welw. Ex Oliv | Native | CU | EM | LC | 1,2,6 | Santos 2916 |
|  | *Utricularia foliosa* L. | Native | CC | FL | LC | 1,2,5,17 |  |
|  | *Utricularia gibba* L. | Native | CC HA HI LA LS MA MO CU | FL | LC | 1,2,5,6,8,17 | Menezes 3459 |
|  | *Utricularia inflexa* Forssk. | Native | CA CN CS CU LA | FL | VU | 1,2,3,5,6,17 | Schlieben 9602 |
|  | *Utricularia livida* E.Mey | Native | HI | EM | LC | 1,2,6 | Barbosa & Moreno 10012 |
|  | *Utricularia paradoxa* F.E. Lloyd & G. Taylor | Native | BE CC HI MO | EM | LC | 1,6 | Young 1421 |
|  | *Utricularia reflexa* Oliv. | Native | BE CC HI LS MO | FL | LC | 1,2,3,5,6,17 | Benso 112 |
|  | *Utricularia simulans* Pilg. | Native |  | A | LC | 1,2,8 |  |
|  | *Utricularia stellaris* L.f. | Native | CS CU LA | EM | VU | 1,2,5 |  |
|  | *Utricularia striatula* Sm. | Native | MA HI CU | EM | LC | 1,2,6 | Gouveia 948/1054/1069/1115 |
|  | *Utricularia subulata* L. | Native | CC HI LS MA MO | EM | LC | 1,2,5,6,8,17 | Chase 2601 |
|  | *Utricularia welwitschi*i Oliv. | Native | HI | EM | LC | 1,2,6 | Bester 9080 |
| **Linderniaceae** | *Lindernia diffusa (L.)* Wettst. | Native |  | EM | LC | 1,2,5 |  |
|  | *Lindernia linearifolia* (Engl.) Eb.Fisch. | Endemic | HI | EM |  | 1,5 |  |
|  | *Lindernia pulchella* (Skan) Philcox | Native | BE | A | LC | 1,18 |  |
|  | *Lindernia senegalensis* Benth. | Native |  | EM | LC | 1,2 |  |
|  | *Torenia thouarsii* (Cham. & Schltdl.) Kuntze | Native |  | EM | LC | 1,2,5 |  |
| **Lythraceae** | *Ammannia auriculata* Willd. | Native | BO CU HI MA NA | EM | LC | 1,2,5 |  |
|  | *Ammannia baccifera* L. | Native | CC HI MA NA ZA | EM | NT | 1,2,5 |  |
|  | *Ammannia prieuriana* Guill. & Perr. | Native | CN MA NA ZA | EM | LC | 1,2,5 |  |
|  | *Ammannia senegalensis* Lam. | Native | CU | EM | LC | 1,2,5 |  |
|  | *Rotala capensis* (Harv.) A.Fern. & Diniz | Native | HI | EM |  | 1,5,15 |  |
|  | *Rotala filiformis* (Bellardi) Hiern | Native | CC CS CU HI MA | EM |  | 1,5,6,15 | Menezes 803 |
|  | *Rotala fluitans* Pohnert | Native | CU | EM | LC | 1,2,5,6,15 | Santos & Barroso 2711 |
|  | *Rotala fontinalis* Hiern | Native | BE MA | EM | DD | 1,2,5 |  |
|  | *Rotala gossweileri* Koehne | Native | BE LS MA | EM | LC | 1,2,5 |  |
|  | *Rotala lucalensis* A.Fern. & Diniz | Native | CS MA | EM |  | 1,5 |  |
|  | *Rotala mexicana* Schltdl. & Cham. | Native | MA | EM | LC | 1,2,5,15 |  |
|  | *Rotala myriophylloides* Welw. ex Hiern | Native | CC HI LS | EM |  | 1,5,6,15 | Menezes 2385; Santos 2257 |
|  | *Rotala nummularia* Welw. ex Hiern | Endemic | CC HI | EM |  | 1,6 | Santos 177; Mendonça 115 |
|  | *Rotala serpiculoides* Welw. ex Hiern | Native | HI | EM |  | 1,5 |  |
|  | *Rotala smithii* A.Fern. & Diniz | Native | ZA | EM | VU | 1,2,5 |  |
|  | *Rotala thymoides* Exell | Endemic | HI | EM |  | 1,5 | Mendes 3602 |
|  | *Rotala welwitschii Exell* | Native | MA | EM | LC | 1,2,5 |  |
|  | *Trapa natans* L. | Native | CS CU HI MA CC | FL | LC | 1,2,5,6, 19 | Gouveia 1273; Finckh & Zigelski 143598 |
| **Malvaceae** | *Urena lobata L.* | Native | CN LA LN MA CA | A | LC | 1,2,6 | Henriques 1394; Mendonça 97; Monteiro et al., 274; Barbosa & Correia 9126; Maiato FM734 |
| **Melastomataceae** | *Antherotoma debilis* (Sond.) Jacq.-Fél. | Native | BE BI CC CS HA HI LS MA MO | EM |  | 1,6 | Barbosa & Correia 8972; Mendes 3106; Santos 2058; Henriques 903; Menezes 2018; Maiato FM712; Maiato et al. FM1873 |
|  | *Dissotis rhinanthifolia* (Brenan) A.Fern. & R.Fern. | Endemic | BI CC LS MO | EM |  | 1,6 | Santos 1850 |
|  | *Dissotis welwitschii* Cogn. | Native | CC HA HI MO | EM |  | 1,7 | Maiato & Daniel FM029; FM107; FM135 |
| **Menyanthaceae** | *Nymphoides aquatica* (J.F. Gmel.) Kuntze |  | HI | FL |  | 1,5,6 | Menezes 1792 |
|  | *Nymphoides brevipedicellata* (Vatke) A.Raynal | Native |  | FL | LC | 1,2,5,15 |  |
|  | *Nymphoides forbesiana* (Griseb.) Kuntze | Native | CC | FL | LC | 1,2,5,6,15 | Maiato & Gomes FM271 |
|  | *Nymphoides indica* (L.) Kuntze subsp. *occidentalis* A.Raynal |  | CC CU HI | FL | LC | 1,2,5,6,9, 15 | Gouveia 1093; Menezes 2313 |
|  | *Nymphoides rautanenii* (N.E.Br.) A.Raynal | Native | HI | FL | LC | 1,2,5,15 |  |
| **Nymphaeaceae** | *Nymphaea divaricata* Hutch. | Native |  | FL | DD | 1,2,5 |  |
|  | *Nymphaea heudelotii* Planch. | Native | BO CC LS MA MO ZA | FL | LC | 1,2,6,7,20 | Barker et al. 44; Goyder 8259 |
|  | *Nymphaea lotus* L. | Native | BO CN LN MA NA ZA | FL | LC | 1,2,5,9 | Barker et al. 44; Goyder 8259 |
|  | *Nymphaea nouchali* Burm.f. var. *caerulea* (Savigny) | Native | BO CC CN HA HI LS MA MO NA | FL | LC | 1,2,5,6, 14,20 | Frisby 4013; Goyder 8296; Goyder 8376 |
|  | *Nymphaea sulphurea* Gilg. | Native | CC LS MO | FL | DD | 1,2,5 |  |
| **Ochnaceae** | *Sauvagesia erecta* L. | Native | LS ZA | A | LC | 1,2,8 |  |
| **Onagraceae** | *Epilobium hirsutum L.* | Native | HI | EM | LC | 1,2,5 |  |
|  | *Ludwigia abyssinica* A. Rich. | Native | HA | EM | LC | 1,2,5,6,15 | Barbosa & Correia 8878 |
|  | *Ludwigia adscendens* (L.) H.Hara | Native | BO HI LN MA NA | EM | LC | 1,2,5,6,15 | Goldsmith 104/60; Balsinhas 521 |
|  | *Ludwigia erecta (L.) H.Hara* | Introduced | BO LA NA | EM |  | 1,3,6,15 | Teixeira & Santos 22 |
|  | *Ludwigia hyssopifolia* (G.Don) Exell | Introduced | CA | EM | LC | 1,3,5,6 | Monteiro & Murta 226 |
|  | *Ludwigia leptocarpa* (Nutt.) H.Hara | Native | HI LA LS | EM | LC | 1,2,5,6,8, 15 | Morais & Pires 1063 |
|  | *Ludwigia octovalvis* (Jacq.) P.H.Raven | Native | BE BI BO CA CC CN CS CU LA LN LS MA MO NA | EM | LC | 1,2,5,6,8,15 | Teixeira & Santos 79; Barbosa 8825; Correia 77/1093 |
|  | *Ludwigia palustris* (L.) Elliott | Native | HI | EM | LC | 1,2,5,6,15 | Menezes 2319 |
|  | *Ludwigia senegalensis* (DC.) Troch. | Native | CU HI | S | LC | 1,2,5,6 | Menezes et al., 3020 |
|  | *Ludwigia stenorraphe* (Brenan) H.Hara | Native | HI MA | FL | LC | 1,2,5 |  |
| **Orobanchaceae** | *Buchnera attenuata* Skan | Native |  | A |  | 1,7 |  |
|  | *Gerardiina angolensis* Engl. | Native |  | A |  | 1,6 | Santos 179; Henriques 970; Daniel 2788; Bergh 1964 |
|  | *Micrargeriella aphylla* R.E.Fr. |  | MO | A |  | 1,7 |  |
|  | *Sopubia simplex* (Hochst.) Hochst. | Native |  | A |  | 1,6 | Menezes 1881; Henriques 670; Goldsmith 82/66 |
| **Plantaginaceae** | *Bacopa crenata* (P.Beauv.) Hepper | Native |  | EM | LC | 1,2,18 |  |
|  | *Bacopa occultans* (Hiern) Hutch. & Dalziel | Native | MA | EM |  | 1,8 |  |
|  | *Dopatrium angolense* Skan | Endemic | HI | EM |  | 1,21 |  |
|  | *Dopatrium caespitosum* P.Taylor | Endemic | MO | EM |  | 1,21 |  |
|  | *Dopatrium stachytarphetoides* Engl. & Gilg | Native | CC | EM |  | 1,5,21 |  |
|  | *Limnophila ceratophylloides* (Hiern) Skan | Native | HI BI | EM |  | 1,5,18 |  |
|  | *Limnophila dasyantha* (Engl. & Gilg) Skan | Native | CC | EM | LC | 1,2 |  |
|  | *Limnophila indica* (L.) Druce | Native | BE | EM | LC | 1,2,5,18 |  |
|  | *Limosella aquatica* L. | Native | HI | EM | LC | 1,2,5,18 |  |
|  | *Mimulus gracilis* R.Br. | Native | HI | EM | LC | 1,2,6,18 | Santos 685 |
| **Podostemaceae** | *Angolaea fluitans* Wedd. | Native |  | EM |  | 1,5 |  |
|  | *Inversodicraea cristata* Engl. | Native |  | EM | VU | 1,2,5,22 |  |
|  | *Inversodicraea digitata* H.E.Hess | Endemic |  | EM |  | 1,5,22 |  |
|  | *Inversodicraea fluitans* H.E.Hess | Endemic |  | EM |  | 1,5,22 |  |
|  | *Inversodicraea ledermannii* (Engl.) Engl. | Native |  | EM | LC | 1,2,5,22 |  |
|  | *Inversodicraea tenax*  C.Cusset | Native |  | EM |  | 1,5,22 |  |
|  | *Inversodicraea warmingiana* (Gilg) Engl. | Native |  | EM | VU | 1,2,5,14,22 |  |
|  | *Ledermanniella aloides* (Engl.) C.Cusset | Native |  | EM | VU | 1,2,5 |  |
|  | *Leiothylax quangensis* (Engl.) Warm. | Native |  | EM | EN | 1,2,5 |  |
|  | *Letestuella tisserantii* G.Taylor | Native |  | EM | LC | 1,2,5,14 |  |
|  | *Saxicolella angola* Cheek | Endemic | CS CN | EM |  | 29 |  |
|  | *Sphaerothylax abyssinica* (Wedd.) Warm. | Native |  | EM | LC | 1,2,5 |  |
|  | *Sphaerothylax algiformis* Bisch. ex C.Krauss | Native | BE | EM | LC | 1,2,5,14 |  |
|  | *Tristicha trifaria* (Bory ex Willd.) Spreng. | Native | CN | EM | LC | 1,2,5,14 |  |
| **Polygonaceae** | *Persicaria decipiens* (R.Br.) K.L.Wilson | Native |  | EM |  | 1,5,23 |  |
|  | *Persicaria glomerata* Dammer) S.Ortiz & Paiva | Native |  | EM |  | 1,5 |  |
|  | *Persicaria limbata* (Meisn.) H.Hara | Native |  | EM | LC | 1,2,5,6,23 | Bergh 1932; Maiato & Gomes FM259 |
|  | *Persicaria nogueirae* S.Ortiz & Paiva | Native |  | EM |  | 1,5 |  |
|  | *Persicaria senegalensis* (Meisn.) Soják | Native |  | EM | LC | 1,2,5,23 |  |
|  | *Polygonum plebeium* R.Br. | Native | HI | EM | LC | 1,2,6 | Daniel 2978 |
|  | *Polygonum tomentosum* Wild. | Native | HI | EM | LC | 1,2,6 | Daniel 2665; Kussumwa 468 |
| **Primulaceae** | *Anagallis kochii* H.E.Hess | Native | HI | EM | NT | 1,2,5 |  |
| **Ranunculaceae** | *Clematis grandiflora* DC. | Native | CA CN CS | EM |  | 1,6 | Daniel 2875; Daniel & Timóteo 3603 |
|  | *Ranunculus multifidus* Forssk. | Native | BE HI MA NA | EM | LC | 1,2,5 |  |
| **Rubiaceae** | *Oldenlandia capensis* L.f. | Native | CU HI LA NA | EM | LC | 1,2,5 |  |
|  | *Oldenlandia lancifolia* (K. Schum.) DC. | Native | CA | EM | LC | 1,2 |  |
| **Sphenocleaceae** | *Sphenoclea zeylanica* Gaertn. | Doubtful |  | EM | LC | 1,2,3,5,6,24 | Barbosa & Carvalho 3757 |
| **Theophrastaceae** | *Samolus valerandi L.* | Native | NA | EM | LC | 1,2,6,14 | Huntley 3568; Ward 110 |
| **MONOCOTYLEDONS** | | | | | | | |
| **Alismataceae** | *Alisma plantago-aquatica*L. | Introduced | HI | EM | LC | 1,2,5,6,14 | Kussumwa 2 |
|  | *Caldesia parnassifolia* Parl. | Native |  | FL | LC | 1,2,5 |  |
|  | *Limnophyton angolense* Buchenau | Native | CC | EM | LC | 1,2,5,6,9,14 | Finckh 132708/132853; Maiato FM1322 |
|  | *Limnophyton obtusifolium* (L.) Miq. | Native |  | EM | LC | 1,2,3,5,14 |  |
|  | *Rasnalisma humile* (Rich. ex Kunth) Hutch. | Native |  | EM | LC | 1,2,5 |  |
|  | *Wiesneria filifolia*Hook.f. | Native |  | EM | LC | 1,2,5 |  |
|  | *Wiesneria schweinfurthii* Hook.f. | Native |  | EM | LC | 1,2,5,14 |  |
| **Amaryllidaceae** | *Crinum binghamii* Nordal & Kwembeya | Native | MO | EM |  | 1,7 |  |
|  | *Crinum macowanii* Baker | Native |  | EM |  | 1,5 |  |
| **Aponogetonaceae** | *Aponogeton abyssinicus Hochst. ex A.Rich.* |  |  | EM | LC | 1,2,5 |  |
|  | *Aponogeton desertorum* *Zeyh. ex Spreng.* | Native | BE | EM | LC | 1,2,5,6,14 | Hankey & Van der Walt 2732 |
|  | *Aponogeton junceus* Lehm. | Native |  | EM | LC | 1,2,5,14 |  |
| **Araceae** | *Anubias hastifolia* Engl. | Native |  | EM | LC | 1,2 |  |
|  | *Anubias heterophylla* Engl. | Native |  | EM | LC | 1,2,5 |  |
|  | *Cercestis congensis* Engl. | Native |  | EM | LC | 1,2 |  |
|  | *Cyrtanthus welwitschii Hiern ex Baker* | Native | CC | EM |  | 1,2,7 |  |
|  | *Lasimorpha senegalensis* Schott | Native |  | EM | LC | 1,2,5 |  |
|  | *Lemna aequinoctialis* Welw. | Native |  | FF | LC | 1,2,5,8,14 |  |
|  | *Pistia stratiotes* L. | Native | HI | FF | LC | 1,2,3,5,6,9,13,25 | Daniel 3676 |
|  | *Wolffia arrhiza* (L.) Horkel ex Wimm. | Native |  | FF | LC | 1,2,3,5,14 |  |
|  | *Wolffia cylindracea* Hegelm. | Native |  | FF | LC | 1,2,5 |  |
|  | *Wolffia globosa* (Roxb.) Hartog & Plas | Introduced |  | FF | LC | 1,2,5,14 |  |
|  | *Wolffiella welwitschii* (Hegelm.) Monod | Native |  | FF | LC | 1,2,5,14 |  |
| **Cannaceae** | *Canna indica* L. var. *indica* | Introduced | UI | EM |  | 1,6,13 |  |
| **Colchicaceae** | *Gloriosa sessiliflora* Nordal & M.G.Bingham | Native | CC MO | EM |  | 7 |  |
| **Commelinaceae** | *Aneilema umbrosum* (Vahl) Kunth | Native | LN | EM |  | 1,13 |  |
|  | *Commelina benghalensis* L. | Native | LA | EM | LC | 1,2,5,6 | Revermann 136467 |
|  | *Commelina diffusa* Burm.f. | Native |  | EM | LC | 1,2,5,6,8, 26 | Finckh 134979 |
|  | *Commelina erecta* L. | Native |  | EM | LC | 1,2,5,13 |  |
|  | *Commelina subulata* Roth. | Native | MA HI | EM | LC | 1,2,5,6,26 | Daniel 3158 |
|  | *Floscopa flavida* C.B.Clarke | Native | CC | EM | LC | 1,2,26 |  |
|  | *Floscopa glomerata* (Willd. ex Schult. & Schult.f.) | Native |  | EM | LC | 1,2,5,26 |  |
| **Cymodoceaceae** | *Halodule wrightii* Asch. | Native |  | EM | LC | 1,2 |  |
| **Cyperaceae** | *Abildgaardia ovata* (Burm.f.) Kral | Native |  |  |  | 1,7,27 |  |
|  | *Bolboschoenus maritimus* (L.) Palla | Native |  | EM | LC | 1,2,5,20,27 |  |
|  | *Bolboschoenus nobilis* (Ridl.) Goetgh. & | Native | NA | EM |  | 1,5,27 |  |
|  | *Bulbostylis densa* (Wall.) Hand.-Mazz | Native | HI | EM | LC | 1,2,5 |  |
|  | *Bulbostylis hispidula* (Vahl) R.W. Haines | Native | CC | EM | LC | 1,2,6,20 | Wallenfang 136451; Finckh & Zigelski 143612 |
|  | *Bulbostylis schoenoides* (Kunth) | Native |  | EM | LC | 1,2,20,27 |  |
|  | *Carex angolensis* Nelmes | Native | MO | EM |  | 1,6 | Archer 3612 |
|  | *Cladium mariscus* (L.) Pohl | Introduced |  | EM | LC | 1,2,5,20,27 |  |
|  | *Cyperus albescens* (Steud.) Larridon & Govaerts | Native |  | EM | LC | 1,2,5,20,27 |  |
|  | *Cyperus albiceps* Ridl. | Native |  | EM | LC | 1,2,27 |  |
|  | *Cyperus abietinus* (Goetgh.) Bauters | Native | BE | EM |  | 1,27 |  |
|  | *Cyperus aethiops* Welw. ex Ridl. | Native |  | EM | LC | 1,2,20,27 |  |
|  | *Cyperus alopecuroides* sensu Thunb | Native | BE | EM | LC | 1,2,5,20,27 |  |
|  | *Cyperus alternifolius* L. | Native | NA | EM | LC | 1,2,5 |  |
|  | *Cyperus amabilis* Vahl. | Native | HI CC | EM | LC | 1,2,5,6 | Daniel 3692; Wallenfang 136450 |
|  | *Cyperus aromaticus* (Ridl.) Mattf. & Kük. | Native |  | EM |  | 1,20,27 |  |
|  | *Cyperus articulatus* L. | Native | HI | EM | LC | 1,2,3,5,20,27 |  |
|  | *Cyperus ascocapensis* Bauters | Native | HA | EM | LC | 1,2,27 |  |
|  | *Cyperus ascopusillus* Goetgh. | Native |  | EM |  | 1,5 |  |
|  | *Cyperus assimilis* Steud. | Native | HI | EM | LC | 1,2,27 |  |
|  | *Cyperus blepharoleptos* Steud. | Native |  | EM | LC | 1,2,3,5,20,27 |  |
|  | *Cyperus brasiliensis* (Kunth) Bauters | Native |  | EM | LC | 1,2,5 |  |
|  | *Cyperus bulbosus* Vahl | Native | LA | EM | LC | 1,2 |  |
|  | *Cyperus chrysocephalus* (K.Schum.) Kük. | Native |  | EM |  | 1,7 |  |
|  | *Cyperus compressus L.* | Native |  | EM | LC | 1,2,5,20 |  |
|  | *Cyperus corymbosus* Rottb. | Native |  | EM | LC | 1,2,5 |  |
|  | *Cyperus cuspidatus* Kunth. | Native | BE | EM |  | 1,5 |  |
|  | *Cyperus cyperoides* (L.) Kuntze | Native | HI | EM | LC | 1,2 |  |
|  | *Cyperus deciduus* Boeckeler | Native | BE | EM |  | 1,2,27 |  |
|  | *Cyperus denudatus* L.f. | Native | HI | EM | LC | 1,2,6,20,27 | Daniel 3533/3761; Bergh 1928 |
|  | *Cyperus dichrostachyus* Hochst. ex A.Rich. | Native |  | EM | LC | 1,2 |  |
|  | *Cyperus difformis* L. | Native | HI | EM | LC | 1,2,5,27 |  |
|  | *Cyperus digitatus* Roxb. | Native | BE | EM | LC | 1,2,5,8,20,27 |  |
|  | *Cyperus distans* L. | Native | HI | EM | LC | 1,2,3,5,6 | Daniel 2906/3768 |
|  | *Cyperus dives* Delile | Native |  | EM | LC | 1,2,5,20,27 |  |
|  | *Cyperus durus* Kunth | Native |  | EM |  | 1,27 |  |
|  | *Cyperus elegantulus* Steud. | Native |  | EM | LC | 1,2,20 |  |
|  | *Cyperus erectus* (Schumach.) Mattf. & Kük. | Native | NA | EM | LC | 1,2,20,27 |  |
|  | *Cyperus esculentus* L. | Native | HI | EM | LC | 1,2,5,6,8 | Daniel 2664/3447 |
|  | *Cyperus exaltatus* Retz. | Native | LA | EM | LC | 1,2,5 |  |
|  | *Cyperus flavescens* L. | Native | HA | EM | LC | 1,2,5,27 |  |
|  | *Cyperus gossweileri* Kük. | Endemic | BE | EM |  | 1,2,20 |  |
|  | *Cyperus haspan* L. | Native | HI | EM | LC | 1,2,5,6 | Nougueira 20; Daniel 3766 |
|  | *Cyperus hensii* T.Durand & Schinz | Native |  | EM |  | 1,7 |  |
|  | *Cyperus hortensis* (Salzm. ex Steud.) Dorr | Native | BE | EM | LC | 1,2 |  |
|  | *Cyperus hystricoides* (B.Nord.) Bauters | Native | HI | EM | LC | 1,2,27 |  |
|  | *Cyperus imbricatus* Retz. | Native | HI | EM | LC | 1,2,5,6,27 | Goyder et al., 7316 |
|  | *Cyperus isolepis* (Nees) Bauters | Native | MA | EM | LC | 1,2,27 |  |
|  | *Cyperus kyllingiella* Larridon | Native |  | EM | LC | 1,2,27 |  |
|  | *Cyperus laevigatus* L. | Native | NA | EM | LC | 1,2,5,6,20,27 | Smook 12129 |
|  | *Cyperus lanceolatus* Poir. | Native | MO | EM | LC | 1,2,5 |  |
|  | *Cyperus latifolius* Poir. | Native |  | EM | LC | 1,2,27 |  |
|  | *Cyperus laxespicatus* Kük. | Native |  | EM | LC | 1,2 |  |
|  | *Cyperus lipofiliformis* Goetgh. | Native |  | EM | LC | 1,2 |  |
|  | *Cyperus longus* L. | Native | NA | EM | LC | 1,2,5,6,20,27 | Ward 107 |
|  | *Cyperus macrostachyos* Lam. | Native |  | EM | LC | 1,2,5,20,27 |  |
|  | *Cyperus marginatus* Thunb. | Native | NA | EM |  | 1,6,20,27 | Huntley et al., 124; Ward 91/78; Smook 12065/12064 |
|  | *Cyperus melanospermus* (Nees) Valck.Sur. | Native |  | EM | LC | 1,2,5,20,27 |  |
|  | *Cyperus melas* Ridl. | Native | HI | EM | LC | 1,2 |  |
|  | *Cyperus metzii* (Hochst. ex Steud.) Mattf. & Kük. | Native |  | EM |  | 1,5 |  |
|  | *Cyperus mundii* (Nees) Kunth | Native | NA | EM | LC | 1,2,5, 20,27 |  |
|  | *Cyperus nigricans* Steud. | Native | HI | EM | LC | 1,2,20,27 |  |
|  | *Cyperus nitidus* Lam. | Native |  | EM | LC | 1,2,5,20,27 |  |
|  | *Cyperus nutans* Vahl | Native |  | EM | LC | 1,2,5 |  |
|  | *Cyperus obtusatus* (J.Presl & C.Presl) Mattf. & Kük. | Native |  | EM |  | 1,5 |  |
|  | *Cyperus odoratus* L. | Native |  | EM | LC | 1,2,5 |  |
|  | *Cyperus papyrus* L. | Native | MO | EM | LC | 1,2,3,5,6,9,20,27 | Maiato FM193; Maiato & Huntley FM145; Maiato & Mumbundu FM549 |
|  | *Cyperus pauper* Hochst. ex A.Rich. | Native |  | EM | LC | 1,2 |  |
|  | *Cyperus pectinatus* Vahl | Native | CN | EM | LC | 1,2,5,6,7,27 | Goyder 8078 |
|  | *Cyperus pelophilus* Ridl. | Native | NA | EM | LC | 1,2,20,27 |  |
|  | *Cyperus persquarrosus* T.Koyama | Native | HI | EM | LC | 1,2,27 |  |
|  | *Cyperus platycaulis* Baker | Native |  | EM | LC | 1,2,5,7 |  |
|  | *Cyperus polystachyos* Rottb. | Native | NA | EM | LC | 1,2,5,20,27 |  |
|  | *Cyperus proteus* (Welw.) Bauters | Native | HI MO | EM | LC | 1,2,6,7 | Daniel & Timóteo 3657; Goyder 8005 |
|  | *Cyperus pumilus* L. | Native |  | EM | LC | 1,2,5,20,27 |  |
|  | *Cyperus pustulatus* Vahl | Native | BE | EM | LC | 1,2 | Daniel 3769 |
|  | *Cyperus renschii* Boeckeler | Native |  | EM | LC | 1,2 |  |
|  | *Cyperus richardii* Steud. | Native |  | EM | LC | 1,2 |  |
|  | *Cyperus ridleyi* Mattf. & Kük. | Native | HI | EM | LC | 1,2,27 |  |
|  | *Cyperus rotundus* L. | Native | LA BO | EM | LC | 1,2,5,6 | Nougueira 60 |
|  | *Cyperus rubicundus* Vahl | Native | BE | EM | LC | 1,2 |  |
|  | *Cyperus ruwenzoriensis* (C.B.Clarke) Huygh | Native |  | EM |  | 1,20,27 |  |
|  | *Cyperus sesquiflorus* (Torr.) Mattf. & Kük. | Native | HI | EM | LC | 1,2,5 |  |
|  | *Cyperus sphacelatus* Rottb. | Native |  | EM |  | 1,5 |  |
|  | *Cyperus squarrosus* L. | Native | NA | EM | LC | 1,2,5 |  |
|  | *Cyperus submicrolepis* Kük. | Native |  | EM | LC | 1,2 |  |
|  | *Cyperus subsquarrosus* (Muhl.) Bauters | Native | HI | EM | LC | 1,2,27 |  |
|  | *Cyperus subtenax* Kük. | Endemic | BE | EM |  | 1,5 |  |
|  | *Cyperus subtrigonus* (C.B.Clarke) Kük. |  |  | EM |  | 7 | Goyder 8940 |
|  | *Cyperus tenuiculmis* Boeckeler | Native | MO | EM | LC | 1,2,5 |  |
|  | *Cyperus tenuiflorus* (Rottb.) Boeck. | Native |  | EM | LC | 1,2 |  |
|  | *Cyperus tenuispica* Steud. | Native |  | EM | LC | 1,2,5,27 |  |
|  | *Cyperus tuberosus* Rottb. | Native |  | EM | LC | 1,2 |  |
|  | *Cyperus xantholepis* (Nelmes) Lye | Native | MO | EM | LC | 1,2,5 |  |
|  | *Cyperus welwitschii* (Ridl.) Lye | Native | MA | EM |  | 1,27 |  |
|  | *Cyperus zollingeri* Steud. | Native |  | EM | LC | 1,2 |  |
|  | *Eleocharis acutangula* (Roxb.) Schult. | Native | HI | EM | LC | 1,2,5,6,20,27 | Bergh 1929 |
|  | *Eleocharis complanata* Boeck | Native |  | EM | LC | 1,2 |  |
|  | *Eleocharis cubangensis* H.E.Hess | Endemic |  | EM | DD | 1,2,3,5 |  |
|  | *Eleocharis dulcis* (Burm.f.) Trin. ex Hensch. | Native | MO | EM | LC | 1,2,3,5,6,27 | Finckh & Zigelski 143263 |
|  | *Eleocharis retroflexa* (Poir.) Urb | Native |  | EM | LC | 1,2,5,27 |  |
|  | *Eleocharis variegata* (Poir.) C.Presl | Native |  | EM | LC | 1,2,5,27 |  |
|  | *Fimbristylis bisumbellata* (Forssk.) Bubani | Native | NA | EM | LC | 1,2,5,20,27 |  |
|  | *Fimbristylis complanata* (Retz.) Link | Native |  | EM | LC | 1,2,5,20,27 |  |
|  | *Fimbristylis dichotoma* (L.) Vahl | Native |  | EM | LC | 1,2,5,20,27 |  |
|  | *Fimbristylis ferruginea* (L.) Vahl | Native |  | EM | LC | 1,2,5,20,27 |  |
|  | *Fimbristylis ovata* (Burm.f.) J.Kern | Native |  | EM | LC | 1,2,5 |  |
|  | *Fimbristylis squarrosa* Vahl | Native | HI | EM | LC | 1,2,5,27 |  |
|  | *Fuirena angolensis* (C.B.Clarke) Lye ex J.Raynal & Roessler | Native | HI | EM |  | 1,27 |  |
|  | *Fuirena ciliaris* (L.) Roxb. | Native |  | EM | LC | 1,2,5,27 |  |
|  | *Fuirena leptostachya* Oliv. var. *leptostachya* | Native |  | EM | LC | 1,2,27 |  |
|  | *Fuirena leptostachya* Oliv. var. *nudiflora* K.Schum. |  |  | EM | LC | 1,2,27 |  |
|  | *Fuirena pubescens* (Poir.) Kunth | Native | HI | EM | LC | 1,2,5,6,20,27 | Daniel 2783; Archer 3644 |
|  | *Fuirena stricta* Steud. | Native | HI | EM | LC | 1,2,20,27 |  |
|  | *Fuirena umbellata* Rottb. | Native | MA | EM | LC | 1,2,3,5,6,8,27 | Barker et al., 136 |
|  | *Hypolytrum heterophyllum* Boeckeler |  |  | EM |  | 5 |  |
|  | *Isolepis fluitans* (L.) R.Br. | Native | HA | EM | LC | 1,2,5,20,27 |  |
|  | *Isolepis natans* (Thunb.) A.Dietr. | Native | HI | EM | LC | 1,2,20,27 |  |
|  | *Isolepis sepulcralis* Steud. | Native | HI | EM |  | 1,20,27 |  |
|  | *Rhynchospora candida* (Nees) Boeck | Native | BI CC | EM | LC | 1,2,6,7,27 | Berker et al., 62 |
|  | *Rhynchospora corymbosa* (L.) Britton | Native |  | EM | LC | 1,2,3,5,27 |  |
|  | *Rhynchospora holoschoenoides* (Rich.) Herter | Native | MO | EM | LC | 1,2,5,6,8,27 | Finckh & Zigelski 143323 |
|  | *Rhynchospora rugosa* (Vahl) Gale | Native | CC | EM | LC | 1,2,5,6 | Berker et al., 65 |
|  | *Rhynchospora triflora* Vahl | Native |  | EM | LC | 1,2 |  |
|  | *Schoenoplectiella articulata* (L.) Lye | Native | CU | EM | LC | 1,2,20,27 |  |
|  | *Schoenoplectiella confusa* (N.E.Br.) J.R.Starr | Native | HI | EM | LC | 1,2,5,20,27 |  |
|  | *Schoenoplectiella corymbosa* (Roth ex Roem. & Schult.) J.R.Starr & Jim.Mejías | Native | HI | EM | LC | 1,2,5,20 |  |
|  | *Schoenoplectiella lateriflora* (J.F.Gmel.) Lye | Native |  | EM | LC | 1,2,5 |  |
|  | *Schoenoplectiella mucronata* (L.) J.Jung & H.K.Choi | Native | HI | EM | LC | 1,2 |  |
|  | *Schoenoplectiella muricinux* (C.B.Clarke) J.R.Starr | Native | HI | EM | LC | 1,2,6,20,27 | Smook 12140 |
|  | *Schoenoplectiella muriculata* (Kük.) J.R.Starr | Native | HI | EM | LC | 1,2,20,27 |  |
|  | *Schoenoplectiella roylei* (Nees) Lye | Native |  | EM | LC | 1,2,5 |  |
|  | *Schoenoplectiella senegalensis* (Steud.) Lye | Native |  | EM | LC | 1,2,20,27 |  |
|  | *Schoenoplectus litoralis* (Schrad.) Palla | Native | NA | EM | LC | 1,2,5,20 |  |
|  | *Scleria bequaertii* De Wild. | Native | MO | EM | LC | 1,2 |  |
|  | *Scleria catophylla C.B.Clarke* | Native |  | EM | LC | 1,2 |  |
|  | *Scleria dregeana* Kunth | Native | HA | EM | LC | 1,2,20,27 |  |
|  | *Scleria erythrorrhiza* Ridl. | Native | HI BI | EM | LC | 1,2,6,7 | Archer 3645; Finckh & Zigelski 132887; Barker et al. 57; Goyder 8933 |
|  | *Scleria flexuosa* Boeck | Native |  | EM | LC | 1,2 |  |
|  | *Scleria foliosa* Hochst. ex A.Rich. | Native | HI | EM | LC | 1,2,20,27 | Daniel 3777 |
|  | *Scleria gracillima* Boeckeler | Native |  | EM | LC | 1,2 |  |
|  | *Scleria greigiifolia* (Ridl.) C.B.Clarke | Native |  | EM | LC | 1,2,5,6,20,27 |  |
|  | *Scleria lagoensis* Boeckeler | Native | HI BI | EM | LC | 1,2,6,27 | Revermann 134265 |
|  | *Scleria lithosperma* (L.) Sw. | Native | HA | EM | LC | 1,2 |  |
|  | *Scleria melanomphala* Kunth | Native | MA | EM | LC | 1,2,6,20,27 | Daniel 3778 |
|  | *Scleria mikawana* Makino | Native |  | EM | LC | 1,2 |  |
|  | *Scleria naumanniana* Boeckeler | Native |  | EM | LC | 1,2 |  |
|  | *Scleria pergracilis* (Nees) Kunth | Native |  | EM | LC | 1,2,20 |  |
|  | *Scleria pooides* Ridl. | Native | MO | EM | LC | 1,2 |  |
|  | *Scleria pulchella* Ridl. | Endemic | HI | EM | CR | 1,2 |  |
|  | *Scleria racemosa* Poir. | Native | UI MO | EM | LC | 1,2,6 | Finckh & Zigelski 143268 |
|  | *Scleria rehmannii* C.B.Clarke | Native |  | EM | LC | 1,2,20,27 |  |
|  | *Scleria tessellata* Willd*.* | Native |  | EM | LC | 1,2,5 |  |
|  | *Scleria welwitschii* C.B.Clarke | Native | BI HI | EM | LC | 1,2,6,20,27 | Archer 1985 |
|  | *Scleria woodii* C.B.Clarke | Native | CU | EM | LC | 1,2,6,20,27 |  |
| **Eriocaulaceae** | *Eriocaulon abyssinicum* Hochst | Native | HI | EM | LC | 1,2,14,20 |  |
|  | *Eriocaulon africanum* Hochst. | Native |  | EM |  | 1,14,20 |  |
|  | *Eriocaulon cinereum* R.Br. | Native |  | EM | LC | 1,2,5,14 |  |
|  | *Eriocaulon lanatum* H.E.Hess | Native | MO | EM |  | 7 | Goyder 8202; Goyder 8369 |
|  | *Eriocaulon latifolium* Sm. | Native |  | EM | LC | 1,2 |  |
|  | *Eriocaulon longipetalum* Rendle | Endemic | HI | EM | DD | 1,2 |  |
|  | *Eriocaulon mutatum* N.E.Br. var. *angustisepalum* | Native |  | EM |  | 1,14,20 |  |
|  | *Eriocaulon schippii* Standl. ex Moldenke | Native | BE | EM |  | 1,5 |  |
|  | *Eriocaulon setaceum* L. | Native |  | EM | LC | 1,2,5,15 |  |
|  | *Eriocaulon teucszii* Engl. & Ruhland | Native | MO | EM |  | 7 | Goyder 8099; Goyder 8364 |
|  | *Eriocaulon transvaalicum* N.E.Br. subsp. *tofieldifoliu*m (Schinz.) | Native |  | EM | LC | 1,2,14,20 |  |
|  | *Eriocaulon transvaalicum* N.E.Br. supsp. *transvaalicum* | Native |  | EM | LC | 1,2,5,14,20 |  |
|  | *Eriocaulon welwitschii* Rendle | Native |  | EM |  | 1,14,20 |  |
|  | *Mesanthemum glabrum* Kimpouni | Native | MO | EM |  | 7 | Baum 645; Frisby 3065; Goyder 8004; Goyder 8201; Goyder 8238; Goyder 8358 |
|  | *Mesanthemum reductum* H.E.Hess | Native |  | EM |  | 7 | Barker et al. 115 |
|  | *Syngonanthus angolensis* H.E.Hess | Native | MO | EM |  | 7 | Goyder 8237; Goyder 8359 |
|  | *Syngonanthus wahlbergii (*Wikstr. ex K rn.) Ruhland var*. wahlbergii* | Native | BE CC HI | EM |  | 1,7,14,20 | Goyder 8100 |
| **Hydrocharitaceae** | *Blyxa hexandra*C.D.K.Cook & Luond | Native | BE | EM | LC | 1,2, 5 |  |
|  | *Blyxa radicans* Ridl. | Native |  | EM |  | 1,5,7 | Baum 827 |
|  | *Lagarosiphon cordofanus*Casp. | Native |  | S | LC | 1,2,5,14 |  |
|  | *Lagarosiphon ilicifolius*Oberm. | Native |  | S |  | 1,5,14 |  |
|  | *Lagarosiphon rubellus* Ridl. | Endemic | HI | S |  | 1,5 |  |
|  | *Najas welwitschii* Rendle | Native |  | S | LC | 1,2,5 |  |
|  | *Ottelia exserta* (Ridl.) Dandy | Native |  | FL | LC | 1,2,5,14 |  |
|  | *Ottelia kunenensis* (Gürke) Dandy | Native | CU | FL | LC | 1,2,5,14 |  |
|  | *Ottelia muricata* (C.H.Wright) Dandy | Native |  | FL | LC | 1,2,5,7,14 | Barker et al. 118 |
|  | *Ottelia ulvifolia* (Planch.) Walp. | Native | HI | FL | LC | 1,2,5,6,7,9,14 | Borges et al., 399; Goyder 8929 |
|  | *Ottelia verdickii* Gürke ex De Wild. | Native |  | FL | LC | 1,2,5 |  |
| **Iridaceae** | *Gladiolus dalenii* Van Geel subsp*. dalenii* | Native | MO HA CS | EM |  | 1,6,7 | Huntley et al., 108; Frisby 3029; Goyder 8461; Maiato FM379; Maiato & Mumbundu FM630/FM697; Goyder et al. 8662; Maiato & Camôngua FM1123; |
|  | *Gladiolus laxiflorus* Baker | Native |  | EM |  | 1,7 | Frisby 3010; Frisby 3066; Goyder & Maiato 8793 |
| **Juncaceae** | *Juncus oxycarpus* E.Mey. ex Kunth | Native | HI | EM | LC | 1,2,5,6,14,20 | Daniel 3094/3757; Daniel & Timóteo 3452; Archer 3643; Smook 12142; Goyder et al., 7458 |
|  | *Juncus punctorius* L.f. | Native |  | EM | LC | 1,2,14,20 |  |
|  | *Juncus rigidus* Desf. | Native |  | EM | LC | 1,2,14,20 |  |
| **Juncaginaceae** | *Triglochin bulbosa* L. | Native |  | EM | LC | 1,2,5,14 |  |
|  | *Triglochin striata* Ruiz & Pav. | Native |  | EM |  | 1,14 |  |
| **Marantaceae** | *Thalia geniculata* L. | Native |  | EM | LC | 1,2,5 |  |
| **Mayacaceae** | *Mayaca baumii* Gürke | Native | BE | EM |  | 1,5,7 | Barker et al. 117; Baum 811 |
| **Orchidaceae** | *Brachycorythis congoensis* Kraenzl. |  |  | EM |  | 1,7 | Frisby 3068 |
|  | *Disa caffra* Bolus | Native | BI | EM |  | 1,7 | Goyder & Maiato 8791 |
|  | *Disa hircicornis* Rchb.f. | Native | HA HI | EM |  | 1,7 | Archer 3610; Frisby 3075 |
|  | *Disa ochrostachya* Rchb. f. |  | HA HI | EM |  | 1,7 | Frisby 4005; Goyder & Maiato 8763; Goyder & Maiato 8796 |
|  | *Disa welwitschii* Rchb.f. | Native | HI | EM |  | 1,7 | Frisby 3063 |
|  | *Eulophia angolensis* (Rchb.f.) Summerh. | Native | HI | EM |  | 1,7 | Frisby 3032 |
|  | *Eulophia horsfallii* (Bateman) Summerh. | Native | MO CS | EM |  | 1,7 | Goyder et al., 8628; Goyder & Maiato 8792 |
|  | *Phaius occidentalis* Schltr. | Native | MO | EM |  | 1,7 | Goyder & Maiato 8761 |
| **Poaceae** | *Acroceras macrum* Stapf | Native | CU HI NA | EM |  | 1,7 |  |
|  | *Agrostis lachnantha* Nees | Native | HI LA | EM | LC | 1,2,5,20 |  |
|  | *Andropogon eucomus* Nees | Native | CC HA HI LS NA | A |  | 1,6,20 | Daniel 2540/2535; Maiato & Daniel FM088 |
|  | *Andropogon huillensis* Rendle | Native | HI | EM |  | 1,2 |  |
|  | *Arundinella nepalensis* Trin | Native | HI MO | EM | LC | 1,2,20 |  |
|  | *Arundo donax* L. | Introduced |  | EM | LC | 1,2,5,20 |  |
|  | *Brachiaria eruciformis* (Sm.) Griseb. | Native | CU HI | EM | LC | 1,2,5 |  |
|  | *Brachiaria mutica* (Forssk.) Stapf | Native |  | EM | LC | 1,2,5 |  |
|  | *Bothriochloa bladhii* (Retz.) S.T. Blake | Native | CC | EM |  | 1,6,20 | Revermann 134482 |
|  | *Chrysopogon nigritanus* (Benth.) Veldkamp | Native | HI | EM | LC | 1,2,5 |  |
|  | *Coix lacryma-jobi* L. | Introduced | UI | A |  | 1,2 |  |
|  | *Cynodon dactylon* (L.) Pers. | Native | BI CC CU HI LA MO | A |  | 1,6 | Daniel & Júnior 2421; Smook 11986; Goyder et al., 7308 |
|  | *Digitaria ciliaris* (Retz.) Koeler | Native | MO | A |  | 1,6 | Revermann 136384/136199 |
|  | *Digitaria longiflora* (Retz.) Pers. | Native | CC | A |  | 1,6 |  |
|  | *Echinochloa colona* (L.) Link. | Native | CC CU NA | EM | LC | 1,2,5,6 | Daniel 2858; Smook 12134 |
|  | *Echinochloa crus-pavonis* (Kunth) Schult. | Native | HI MA | EM | LC | 1,2,5 |  |
|  | *Echinochloa jubata* Stapf | Native | CC MO | EM |  | 1,5,20 |  |
|  | *Echinochloa pyramidalis* (Lam.) Hitchc. & Chase | Native | CC CU HI MO | EM | LC | 1,2,3,5,9,20 | Daniel & Timóteo 3473 |
|  | *Echinochloa stagnina* Retz. P. Beauv. | Native | CU HI | EM | LC | 1,2,3,5,6 | Daniel 2563/2904/3727 |
|  | *Eleusine coracana* (L.) Gaertn. | Native | CC | EM | LC | 1,2,7 | Baum 693 |
|  | *Eleusine indica* (L.) Gaertn | Native | CU MA MO | A | LC | 1,2,6 | Daniel 2554 |
|  | *Elytrophorus globulari*s Hack. | Native | CU HI MO | EM |  | 1,5 |  |
|  | *Eragrostis japonica* (Thunb.) Trin. | Native | HI MO NA | A | LC | 1,2 | Daniel 2857 |
|  | *Hemarthria altissima* (Poir.) Stapf & C.E.Hubb | Native | MO | EM | LC | 1,2,20 |  |
|  | *Hemarthria natans* Stapf | Native | MO | EM |  | 1,5 |  |
|  | *Heteranthoecia guineensis* (Franch.) Robyns | Native | LS | EM | LC | 1,2,5 |  |
|  | *Imperata cylindrica* (L.) Raeusch | Native | CC CN CS HI LA LS MA | EM | LC | 1,2,20 | Maiato 391 |
|  | *Ischaemum fasciculatum* Brongn. | Native | HI | EM | LC | 1,2,20 |  |
|  | *Leersia hexandra* Sw. | Native | MO | EM | LC | 1,2,3,5,7,20 | Goyder 8930 |
|  | *Leptochloa caerulescens* Steud. | Native | MA | EM | LC | 2,5 |  |
|  | *Leptochloa fusca* (L.) Kunth | Native | MO | EM | LC | 1,2,5,20 |  |
|  | *Loudetia angolensis* C.E.Hubb. | Native | MO | EM |  | 1,6,7 | Revermann 134779/134236; Goyder 8264; Maiato FM930 |
|  | *Miscanthus capensis* (Nees) Anderssont | Native |  | EM |  | 1,2 |  |
|  | *Miscanthus junceu*s (Stapf) Pilg. | Native | CC CN MO | EM |  | 1,5,7,9 | Goyder 8299 |
|  | *Oryza barthii* A.Chev | Native | CU HI | EM | LC | 1,2,5,20 |  |
|  | *Oryza longistaminata* A.Chev. & Roehr. | Native | CC MO | EM | LC | 1,2,5,9 | Maiato FM324 |
|  | *Oryzidium barnardii* C.E.Hubb. & Schweick. | Native | CC HI | EM | LC | 1,2 |  |
|  | *Panicum brazzavillense* Franch. | Native | MA | EM | LC | 1,2 |  |
|  | *Panicum coloratum* L. var*. coloratum* | Native | BI CC CN CU HI | EM | LC | 1,2,20 | Smook 12001 |
|  | *Panicum fluviicola* Steud. | Native | CN MA | A | LC | 1,2 | Huntley 3472 |
|  | *Panicum grandiflorum* Trin. ex Nees | Native | MO | A | LC | 1,2 |  |
|  | *Panicum hymeniochilum* Nees | Native | MO | EM | LC | 1,2,20 |  |
|  | *Panicum laetum* Kunth. | Native | MO | A | LC | 1,2 |  |
|  | *Panicum maximum Jacq.* | Native | CC CU | EM |  | 1,3,5,6 | Daniel 166a/2865; Revermann 136276/136197 |
|  | *Panicum parvifolium* Lam. | Native | HI | EM | LC | 1,2,5 |  |
|  | *Panicum repens L.* | Native | CC CU HI | EM | LC | 1,2,5,6 | Smook 12046; Daniel 2815 |
|  | *Panicum subalbidum* Kunth. | Native | MO | EM | LC | 1,2,5 |  |
|  | *Paratheria prostrata* Griseb. | Native | MO | EM | LC | 1,2,5 |  |
|  | *Paspalidium geminatum* (Forssk.) Stapf | Native | HI MO NA | EM | LC | 1,2,5,6 | Ward 90; Smook 12066 |
|  | *Paspalum conjugatum* P.J. Bergius | Introduced | CA LA | EM | LC | 1,2,6 |  |
|  | *Paspalum scrobiculatum* L. | Native | CA CN CU HI | EM | LC | 1,2,5,6,20 | Smook 11958; Maiato FM436 |
|  | *Paspalum urvillei* Steud. | Introduced | HA | EM |  | 1,2 | Daniel 3041 |
|  | *Paspalum vaginatum* Sw. | Native | CS | EM | LC | 1,2,5,20 |  |
|  | *Pennisetum macrourum* Trin. | Native | BO CN CS MA | EM | LC | 1,2,5,20 |  |
|  | *Pennisetum natalense* Stapf | Native |  | EM | LC | 1,2,20 |  |
|  | *Pennisetum purpureum* Schumach. | Native | BO HA HI UI | EM | LC | 1,2,9 | Daniel 2996/2934 |
|  | *Pennisetum thunbergii Thunb.* | Native | HI | EM | LC | 1,2,20 | Smook 12045 |
|  | *Phragmites australis* (Cav.) Steud | Native | CC CU | EM | LC | 1,2,5,20 |  |
|  | *Phragmites mauritianus* Kunth. | Native | CC CU HI LS MO NA | EM | LC | 1,2,5,6,7,9,20 | Daniel 3020/2844; Revermann 134973; Goyder et al., 7385; Goyder 8935; Maiato FM299 |
|  | *Polypogon viridis* (Gouan) Breistr | Introduced | HI | EM | LC | 1,2,6,20 | Goyder et al., 7461 |
|  | *Rhytachne rottboellioide*s Desv | Native | HI | EM | LC | 1,2,6,20 | Smook 12033 |
|  | *Sacciolepis africana* C.E.Hubb. & Snowden | Native | CN CU HI | EM | LC | 1,2,5 |  |
|  | *Sacciolepis indica* (L.) Chase | Native | HI MO | EM | LC | 1,2,5 |  |
|  | *Setaria sphacelata* (Schumach.) Stapf & C.E.Hubb. ex M.B.Moss | Native | CC CN HI MO | EM | LC | 1,2,6,20 | Daniel 3006/2876/3751; Smook 11991; Finckh & Zigelski 143590; Finckh 135599 |
|  | *Sporobolus consimilis* Fresen. | Native | NA | A | LC | 1,2,6 | Ward 111; Huntley 3612; Smook 12073A |
|  | *Sporobolus pyramidalis* P.Beauv. | Native | CC CU MO | EM |  | 1,6,20 | Smook 11981 |
|  | *Sporobolus virginicu*s (L.) Kunth | Native | NA | A | LC | 1,2,6 | Huntley 3502; Smook 12122 |
|  | *Schizachyrium brevifolium* (Sw.) Nees ex Buse | Native | CN HI MO | A | LC | 1,2 |  |
|  | *Schizachyrium sanguineum* (Retz.) Alston | Native | CC CU HI | A |  | 1,6 | Smook 12036; Revermann 136513/ 134531; Maiato FM1312 |
|  | *Vossia cuspidata* Griff. | Native | CC | EM | LC | 1,2,3,5,9 |  |
| **Pontederiaceae** | *Heteranthera callifolia* Rchb. ex Kunth | Native |  | EM | LC | 1,2,5,14 |  |
|  | *Monochoria africana (Solms) N.E.Br.* | Native |  | EM | LC | 1,2,5 |  |
|  | *Pontederia africana* (Solms) M.Pell. & C.N.Horn | Native |  | EM | LC | 1,2,14 |  |
|  | *Pontederia diversifolia* (Vahl) M.Pell. & C.N.Horn | Introduced |  | FL |  | 1,5,8 |  |
|  | *Pontederia crassipes Mart* | Introduced |  | FF |  | 1,5,6 | Daniel & Timóteo 3607 |
| **Potamogetonaceae** | *Potamogeton nodosus* Poir. | Native |  | FL | LC | 1,2,5 |  |
|  | *Potamogeton octandrus* Poir. | Native |  | FL | LC | 1,2,3,5,14 |  |
|  | *Potamogeton polygonifolius* Pourr. |  |  | FL | LC | 1,2,9 |  |
|  | *Potamogeton richardii* Solms | Doubtful |  | FL | LC | 1,2,5 |  |
|  | *Stuckenia pectinata* (L.) Börner | Native |  | S | LC | 1,2,5 |  |
| **Ruppiaceae** | *Ruppia maritima* L. | Native |  | S | LC | 1,2,3,5,14 |  |
| **Typhaceae** | *Typha capensis* (Rohrbach) N.E. Brown | Native |  | EM | LC | 1,2,5,9,14,20 |  |
|  | *Typha domingensis* Persoon | Native |  | EM | LC | 1,2,5,14,20 |  |
| **Xyridaceae** | *Xyris anceps* Lam. | Native |  | EM | LC | 1,2,5 |  |
|  | *Xyris capensi*s Thunberg | Native |  | EM | LC | 1,2,7,14,20 | Goyder 8373 |
|  | *Xyris congensis* Büttner | Native | HI | EM | LC | 1,2,6,7,14,20 | Goyder et al., 7467/7466; Barker et al. 64; Goyder 8322 |
|  | *Xyris foliolata* L.A.Nilsson | Native |  | EM | LC | 1,2,7 | Barker et al. 128 |
|  | *Xyris friesii* Malme | Native | MO | EM | LC | 1,2,7 | Goyder & Maiato 8800 |
|  | *Xyris huillensis* Rendle | Native | HI | EM |  | 1,2 |  |
|  | *Xyris imitatrix* Malme | Native |  | EM | DD | 1,2,7 | Goyder 8332 |
|  | *Xyris obscura* N.E. Br | Native | HI | EM | LC | 1,2,6,14,20 | Finckh & Zigelski 143283 |
|  | *Xyris rehmannii* Nilss | Native | HI MO | EM | LC | 1,2,6,14,20 |  |
